# PTP1B Inhibitory Secondary Metabolites from an Antarctic Fungal Strain *Acremonium* sp. SF-7394

**DOI:** 10.3390/molecules26185505

**Published:** 2021-09-10

**Authors:** Hye Jin Kim, Xiao-Jun Li, Dong-Cheol Kim, Tai Kyoung Kim, Jae Hak Sohn, Haeun Kwon, Dongho Lee, Youn-Chul Kim, Joung Han Yim, Hyuncheol Oh

**Affiliations:** 1 Institute of Pharmaceutical Research and Development, College of Pharmacy, Wonkwang University, Iksan 54538, Korea; mn1003@naver.com (H.J.K.); lixiaojun2017@yahoo.com (X.-J.L.); kimman07@hanmail.net (D.-C.K.); yckim@wku.ac.kr (Y.-C.K.); 2 Division of Polar Life Sciences, Korea Polar Research Institute, Incheon 21990, Korea; tkkim@kopri.re.kr; 3 College of Medical and Life Sciences, Silla University, Busan 46958, Korea; jhsohn@silla.ac.kr; 4 Department of Plant Biotechnology, College of Life Sciences and Biotechnology, Korea University, Seoul 02841, Korea; haeun9906@daum.net (H.K.); dongholee@korea.ac.kr (D.L.)

**Keywords:** *Acremonium* sp., terpenoids, Antarctic fungal metabolites, PTP1B

## Abstract

Chemical investigation of the Antarctic lichen-derived fungal strain *Acremonium* sp. SF-7394 yielded a new amphilectane-type diterpene, acrepseudoterin (**1**), and a new acorane-type sesquiterpene glycoside, isocordycepoloside A (**2**). In addition, three known fungal metabolites, (−)-ternatin (**3**), [D-Leu]-ternatin (**4**), and pseurotin A (**5**), were isolated from the EtOAc extract of the fungal strain. Their structures were mainly elucidated by analyzing their NMR and MS data. The absolute configuration of **1** was proposed by electronic circular dichroism calculations, and the absolute configuration of the sugar unit in **2** was determined by a chemical method. The inhibitory effects of the isolated compounds on protein tyrosine phosphatase 1B (PTP1B) were evaluated by enzymatic assays; results indicated that acrepseudoterin (**1**) and [D-Leu]-ternatin (**4**) dose-dependently inhibited the enzyme activity with IC_50_ values of 22.8 ± 1.1 μM and 14.8 ± 0.3 μM, respectively. Moreover, compound **1** was identified as a competitive inhibitor of PTP1B.

## 1. Introduction

**Figure 1:**
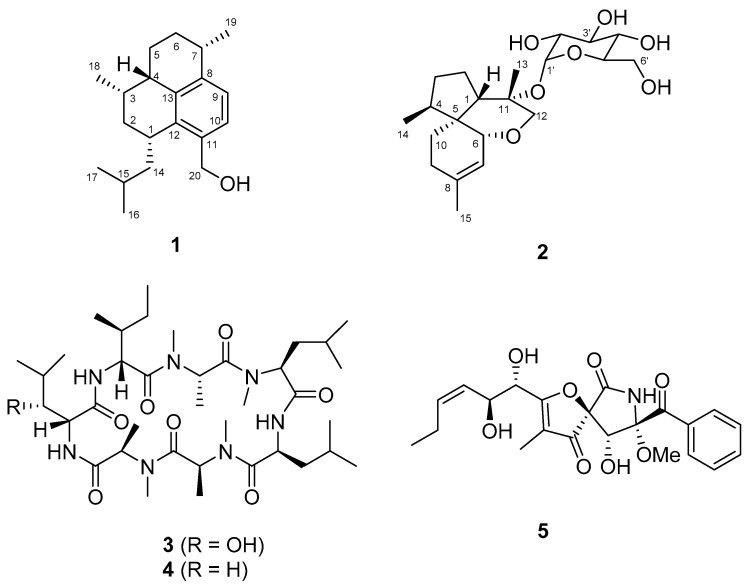
Chemical structures of compounds **1**–**5**.

The fungal genus *Acremonium* has been identified in various sources such as soil, plants, and marine organisms [1]. Secondary metabolites derived from *Acremonium* species have been reported to contain steroids, terpenoids, meroterpenoids, polyketides, alkaloids, and peptides, which show a variety of biological activities, especially anti-bacterial, anti-fungal, and cytotoxic effects [1]. Some examples of these biologically active metabolites include cephalosporin antibiotics [2], cytotoxic heptelidic acid chlorohydrin [3], anti-fungal acremoxanthone A [4], and antibiotic cephaibol B [5]. In the course of our ongoing search for bioactive secondary metabolites from Antarctic-derived fungal strains [6,7], the EtOAc extract obtained from cultures of *Acremonium* sp. SF-7394 was subjected to several separation steps such as column chromatography (CC) using Sephadex LH-20, reversed phase (RP) C_18_, and silica gel as a stationary phase, and RP C_18_ preparative HPLC. As a result, two novel fungal metabolites including an amphilectane-type diterpene, acrepseudoterin (**1**), and an acorane-type sesquiterpene glycoside, isocordycepoloside A (**2**), were isolated. Additionally, three known fungal metabolites including two cyclic heptapeptides, (−)-ternatin (**3**) [8], [D-Leu]-ternatin (**4**) [9], and pseurotin A (**5**) [10] were identified (Figure 1). The evaluation of the inhibitory effects of the isolated compounds on the enzyme activity of protein tyrosine phosphatase 1B (PTP1B) was also conducted in this study. Details of the isolation, structure elucidation, and biological evaluation of these compounds are described herein.

## 2. Results and Discussion

**Figure 2:**
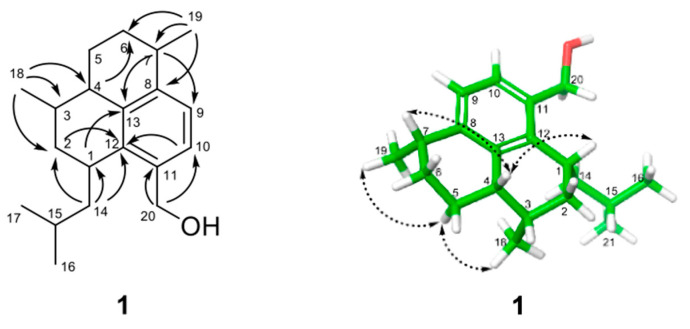
Key HMBC (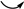) and NOESY (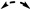) correlations for **1**. The energy-minimized 3D structure was obtained by Macromodel (Version 12.5, Schrodinger LLC) program.

The HRESIMS of compound **1** exhibited a sodium adduct ion at *m/z* 309.2196 [M + Na]^+^, indicating a molecular formula of C_20_H_30_O (calcd for C_20_H_30_ONa, 309.2194) and therefore the presence of six degrees of unsaturation in the molecule. Analysis of the ^1^H, DEPT, and ^13^C NMR spectra of **1** (Table 1) revealed the presence of a 1,2,3,4-tetrasubstituted aromatic ring, four methyl groups (all were doublets in the ^1^H NMR spectrum), five methylene units (one of which was oxygenated), and five methines. This information accounted for four units of unsaturation, suggesting that the compound must possess two additional ring systems. A detailed analysis of the 2D NMR data was next performed to establish the planar structure of **1**. The direct connectivity between hydrogens and carbons in the molecule was established by analyzing the HMQC data, and the spin systems in the molecule were defined by interpreting the COSY data. The COSY spectrum of **1** indicated that the two methyl groups (CH_3_-16 and CH_3_-17) were correlated with the methine proton, H-15; this methine proton was further connected to the methylene protons H_2_-14 that in turn showed a correlation with the methine proton at δ_H_ 3.35 (H-1). Analysis of the COSY data further extended the sequential spin system from H-1 to H-7; the two methyl groups, CH_3_-18 and CH_3_-19, were located at C-3 and C-7, respectively, based on COSY correlations between the respective protons. In addition, the presence of an isolated oxy-methylene unit (H_2_-20) and two mutually ortho-coupled aromatic hydrogens were evident in the COSY data. The connectivity between these spin systems and quaternary carbons was established by analyzing the HMBC spectrum (Figure 2). HMBC correlations of the aromatic hydrogens H-9 and H-10 with the carbons in the aromatic/olefinic carbon region allowed the complete assignment of carbon chemical shifts for the 1,2,3,4-tetrasubstituted benzene ring. Moreover, the HMBC correlation of H-10 to the oxygenated methylene carbon (C-20) connected this carbon to C-11. HMBC correlations of H-7 with C-8 and C-9 and H-4 with C-8 and C-13 provided the connection between C-7 and C-8, and between C-4 and C-13, respectively. Finally, the HMBC correlation of H-2 with C-12 provided evidence for the connection between C-12 and the only remaining connection site C-1. Therefore, the tricyclic planar structure of **1** was assigned as shown to possess a hexahydro-1*H*-phenalene ring system. The carbons in compound **1** were numbered according to the precedent pseudopterosins [11]. The structure of **1** is recognized as a new amphilectane-type diterpenoid [12,13], and the name acrepseudoterin is proposed for compound **1**.

**Figure 3:**
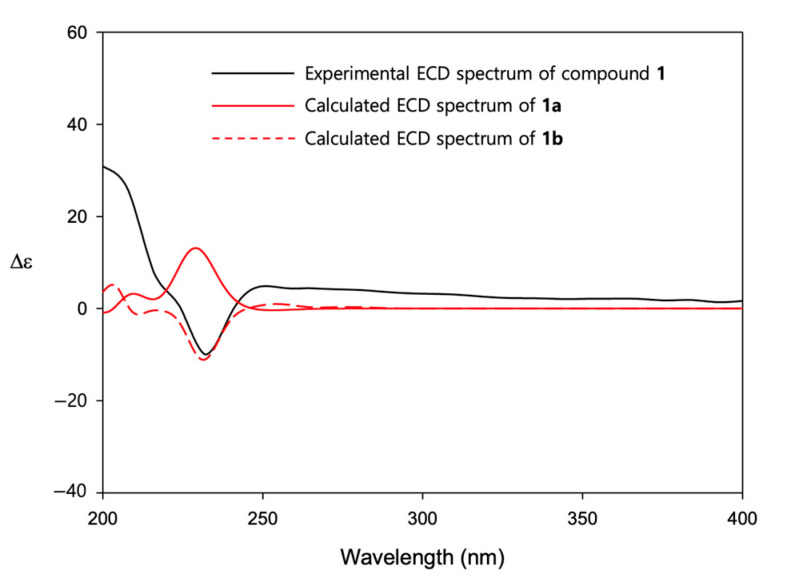
Comparison of calculated and experimental ECD spectra of **1**.

The relative and absolute configuration of **1** was determined by analyzing the NOESY data (Figure 2) and electron circular dichroism (ECD) calculations (Figure 3). The NOESY spectrum of **1** showed correlations of H-4 with H-1 and H-7, indicating that these protons are positioned on the same face of the ring system. On the other hand, NOESY correlations of H-5α with H_3_-18 and H_3_-19 demonstrated the placement of these methyl groups on the opposite face of the ring system, addressing the relative configuration of all the stereocenters in the molecule. The absolute configuration of **1** was proposed by TDDFT (time-dependent density functional theory) computational calculations. The experimental ECD (electronic circular dichroism) spectrum for **1** showed a positive Cotton effect near 250 nm and a negative Cotton effect near 230 nm. After the geometry optimization of each enantiomer of **1** to obtain minimum energy conformers, TDDFT calculated the ECD spectra for the enantiomers; (1*S*, 3*R*, 4*R*, 7*R*)-**1** (**1a**) and (1*R*, 3*S*, 4*S*, 7*S*)-**1** (**1b**) were generated. Comparison of the experimental and calculated ECD spectra indicated that the experimental ECD curve of **1** was almost identical to that calculated for **1b** (Figure 3), suggesting a 1*R*, 3*S*, 4*S*, 7*S* absolute configuration for **1**.

Amphilectane-type diterpenoids possess a structurally interesting tricyclic carbon skeleton and are divided into two groups based on their shared structural characteristics. The pseudopterosin group possesses a tricyclic ring system containing an aromatic ring, while the amphilectane groups possesses a perhydrophenalene ring [13]. Amphilectane-type diterpenoids belonging to the pseudopterosin group such as pseudopterosins [11], helioporin E [14], and pseudopteroxazole [15] generally possess a fully substituted benzene ring and an isobutenyl group. In addition, most compounds of this class were isolated from marine invertebrates, as monoglycosylated forms with various biological activities such as anti-cancer, anti-malarial, and anti-inflammatory effects [11,13]. However, to the best of our knowledge, amphilectane-type diterpenoids with a tetrasubstituted benzene ring, as in **1**, have not been reported yet. Therefore, compound **1** represents a new amphilectane-type diterpenoid of fungal origin possessing unique structural features.

The molecular formula of compound **2** (C_21_H_34_O_7_) was determined on the basis of its HRESIMS and NMR data, indicating five units of unsaturation. Analysis of ^1^H, ^13^C, and DEPT NMR data of **2** (Table 2) indicated the presence of three methyl groups, six methylene units (two of which are oxygenated), eight sp^3^ methines (six of which are oxygenated including one acetal), one sp^2^ methine, and three quaternary carbons (one of which is sp^2^ carbon). As only the resonances for a trisubstituted olefin (δ 121.5 and δ 141.2) were observed in the NMR data to account for unsaturation units, it was deduced that the compound must possess four rings to fulfill the required units of unsaturation. There were nine ^1^H NMR signals in the region for oxygenated sp^3^ hydrogens. This observation, together with five sp^3^ oxygenated methines, including one acetal (δ 95.4, δ 75.6, δ 74.5 δ 74.1 and δ 72.1) and one oxygenated methylene (δ 62.9) signal in the ^13^C NMR spectrum, were suggestive of a hexose moiety. This was also supported by contiguous COSY correlations of five oxymethines and one oxymethylene. Further chemical shift considerations and analysis of coupling constants among respective oxymethines (Table 2), along with the HMBC correlation of H-1′ with C-5′ confirmed the presence of a glucopyranose unit in the molecule. Moreover, it was indicated that the aglycone of **2** must possess no exchangeable hydrogens because the glucose unit accounts for all the exchangeable hydrogens required by the molecular formula.

**Figure 4:**
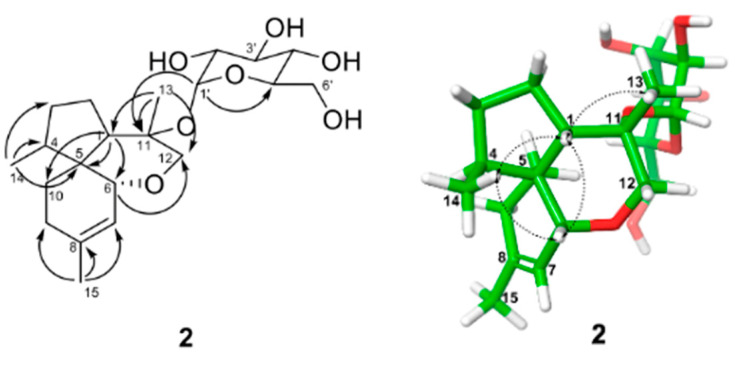
Key HMBC (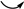) and NOESY (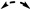) correlations for **2**. The energy-minimized 3D structure was obtained by Macromodel (Version 12.5, Schrodinger LLC) program.

Further structural elucidation of the aglycone of **2** was carried out by analysis of COSY, HMQC, and HMBC data. Detailed analysis of COSY and HMBC data confirmed the presence of a glucopyranose ring moiety in the molecule. Spin systems consisting of H-1-H_2_-2-H_2_-3-H-4-H_3_-14, H-6-H-7-C-8-H_3_-15, and H_2_-9-H_2_-10 were identified by analyzing the COSY data. Connections of these spin systems with the isolated oxygenated methylene unit and quaternary carbons of the aglycone moiety in **2** were established by analyzing the HMBC data (Figure 4). The spin system H-1-H_2_-2-H_2_-3-H-4-H_3_-14 was further extended by HMBC correlations from H_3_-14 to C-5 and from H-2 to C-5, establishing the cyclopentane ring in the molecule. Similarly, the spin systems H_2_-9-H_2_-10 and H-6-H-7-C-8-H_3_-15 were connected via C-8 and C-9 linkage by HMBC correlation from H_3_-15 with C-9, and this unit was further extended to form a cyclohexene ring system on the basis of the HMBC correlations of H_2_-9 and H-7 with C-5. Furthermore, HMBC correlations of H-1 with C-6, H_2_-10 with C-4, and H-6 with C-1 supported the spiro-connection between the aforementioned cyclopentane and cyclohexene moieties, with C-5 being the spiro center. HMBC correlations from the singlet signal H_3_-13 with C-1, C-11, and C-12 led to the connection of the methyl group C-13 to the quaternary carbon C-11 and a connection between C-1 and C-11. Finally, HMBC correlations of H_2_-12 with C-1, C-6, and C-11 led to the assignment of the tetrahydropyran ring moiety in the molecule. Taken together, the aglycone moiety of **2** was assigned to a spiro[4.5]decane system with an additional six-membered ether ring, and the glucopyranose unit was located at C-11 based on the HMBC correlation of the anomeric proton H-1′ with C-11, thus completing the planar structure of **2** as shown.

The relative configuration of the aglycone moiety of **2** was proposed based on the analysis of NOESY correlations (Figure 4). The signal for H-1 correlated with H-6, H_3_-13, and H_3_-14, placing them on the same face of the molecule. Furthermore, NOESY cross-peaks between H-6 and H_3_-14 supported this assignment. Therefore, the relative configuration of **1** for the aglycone portion of the molecule was established as shown. The glucose unit was assigned to be connected through an α-linkage based on the coupling constant (*J* = 3.6 Hz) of the anomeric proton (δ 5.57). To address the absolute configuration of the glucose unit in the molecule, HPLC analysis of the diastereomeric thiocarbamoyl–thiazolidine derivatives was performed [16]. In this analysis, compound **1** was hydrolyzed by heating in 2 M HCl and neutralized with NH_4_OH. After drying in vacuo, the residue was dissolved in pyridine and derivatized with L-cysteine methyl ester and phenyl isothiocyanate, as described in the Experimental section. Direct HPLC analysis of the reaction mixture exhibited the peak with a retention time at 38.0 min, corresponding to that of the thiocarbamoyl–thiazolidine derivative of D-glucose (Supplementary Materials Figure S26). As the anomeric carbon C-1′ is connected to C-11 via ether linkage, and there is free rotation around this bond, the absolute configuration of the aglycone moiety of the molecule could not be assigned with confidence.

The aglycone structure of **2** has the same carbon skeleton as cordycepol A, which was reported as an unusual spiro[4.5]decane sesquiterpene (acorane skeleton) with an additional pyran ring system [17]. Hence, the originally proposed relative configurations at C-1 and C-12 of cordycepol A have been revised [18]. However, the relative configurations of C-1, C-5, and C-6 in compound **2** neither matched with those in the originally proposed nor of revised cordycepol A. In addition, glycosylated cordycepol A has not been reported to date. Therefore, compound **2** was identified as a unique acorane-type sesquiterpene, and it was named isocordycepoloside A.

The structures of the remaining three known compounds, (−)-ternatin (**3**), [D-Leu]-ternatin (**4**), and pseurotin A (**5**) were also elucidated by analyzing the NMR and MS data (Supplementary Materials Figures S19–S25), along with comparisons of the data with those in the literature [8,9,10].

**Figure 5:**
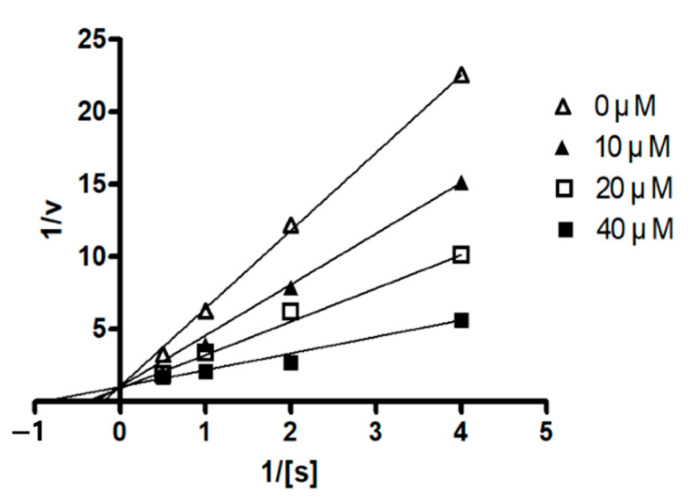
Lineweaver–Burk plots for the inhibition of PTP1B by **1**. The data describe the mean values of three experiments.

To evaluate the biological effects of the compounds isolated in this study, the inhibitory effects against PTP1B activity were investigated. PTP1B has been recognized as a key negative regulator of insulin and leptin pathways and thus has become a new drug target for the treatment of type 2 diabetes mellitus and obesity [19]. In an enzymatic assay using a *p*-nitrophenol phosphate (*p*NPP) as an enzyme substrate, compounds **1** and [D-Leu]-ternatin (**4**) inhibited the PTP1B activity in a dose-dependent manner with IC_50_ values of 22.8 ± 1.1 μM and 14.8 ± 0.3 μM, respectively. The inhibitory effects of the remaining compounds were less pronounced, displaying 12–44% inhibition rates at a concentration of 80 μM. In this assay, ursolic acid (IC_50_ = 3.8 ± 0.5 μM) was employed as the positive control. Following this, we investigated the effect of compound **1** on the kinetic profile of PTP1B-catalyzed pNPP hydrolysis. PTP1B was incubated with different concentrations of *p*-NPP in the absence or presence of compound **1**. The assay was performed using the same method as the PTP1B assay, and the full velocity curves were determined. Kinetic analysis revealed that the inhibition mode of compound **1** was a competitive mode as the Lineweaver–Burk plot showed an increase in *K*_m_, without changing the *V*_max_ value (Figure 5). This result indicates that compound **1** might bind to the active site within PTP1B.

## 3. Materials and Methods

### 3.1. General Experimental Procedures

ECD spectra were recorded on a Jasco J-1100 spectropolarimeter (Jasco Corp., Tokyo, Japan). The UV spectrum was recorded on a UV-vis spectrophotometer (Mecasys, Daejeon, Korea). IR spectra were obtained on a 630 FT-IR spectrometer (Agilent, Palo Alto, CA, USA). Optical rotations were recorded using a Jasco p-2000 digital polarimeter (Jasco Corp., Tokyo, Japan). HRESIMS data were obtained using an AB Sciex Triple TOF 4600 instrument (AB Sciex Pte. Ltd., Framingham, MA, USA). NMR spectra (1D and 2D) were recorded in CD_3_OD (δ_H_/δ_C_ = 3.31/49.0) and pyridine-*d*_5_ (δ_H_/δ_C_ = 7.22, 7.58, 8.74/123.9, 135.9, 150.2) with a Jeol JNM ECP-400 spectrometer (Jeol Ltd., Tokyo, Japan), and the chemical shifts were referenced relative to the residual solvent peaks. HMQC and HMBC experiments were optimized for ^1^*J*_CH_ = 140 Hz and ^n^*J*_CH_ = 8 Hz, respectively. Thin layer chromatography (TLC) was performed on Kieselgel 60 F_254_ (Merck, Darmstadt, Germany) or RP-18 F_254s_ (Merck, Darmstadt, Germany) plates. Spots were visualized by spraying plates with 10% aqueous sulfuric acid (H_2_SO_4_) solution, followed by heating. CC was performed on silica gel (Kieselgel 60, 70–230 mesh, and 230–400 mesh, Merck, Darmstadt, Germany) or YMC octadecyl-functionalized silica gel (75 μm particle size, YMC Co., Ltd., Kyoto, Japan). All preparative-HPLC (prep-HPLC) was operated on a Shiseido prep C_18_ column (20 × 150 mm; 5 μm particle size, ShiseodoCo., Ltd., Tokyo, Japan) with a flow rate of 5 mL/min, and compounds were detected using an ultraviolet detector (absorption at 210 and 254 nm) or an evaporative light scattering detector.

### 3.2. Specimen Collection and Identification

The fungal strain *Acremonium* sp. SF-7394 was isolated from an unidentified lichen collected from the Marian Cove (62°13′09.16″ S, 58°45′57.67″ W) on King George Island, Antarctica in January, 2017. Voucher specimens (SF-7394) have been deposited in the Korea Polar Research Institute. One gram of the sample was mixed with sterile seawater (10 mL), and a portion (0.1 mL) of the sample was processed according to the spread plate method in potato dextrose agar (PDA) medium containing sterile seawater. The isolates were cultured (at 25 °C for 14 days) several times to obtain the final pure culture, and the selected cultures were preserved at −70 °C. The identification of the fungal strain SF-7394 was conducted by analyzing the ITS gene sequence. A GenBank search with the ITS sequence of SF-7394 (GenBank accession number MK307778) indicated *Acremonium rutilum* (NR_077124), *Acremonium cereale* (AB540571), and *Acremonium persicinum* (NR_131260) as the closest matches showing sequence identities of 93.63%, 88.81%, and 88.5%, respectively. Therefore, the fungal strain SF-7394 was characterized as *Acremonium* sp.

### 3.3. Isolation of Compounds ***1***–***5***

The fungal strain SF-7394 was cultured in 23 fernbach flasks (1 L), each containing 200 mL of potato dextrose agar (PDA) medium with 3% NaCl, for 28 days at 25 °C. The combined PDA media was extracted with EtOAc (23 L). The combined extracts were filtered through filter paper and evaporated to dryness, resulting in a crude extract, SF-7394 (2.5 g). The crude extract was fractionated on RP C_18_ flash CC (4.5 × 30 cm), eluted with a stepwise gradient of 20, 40, 60, 80, and 100% (*v/v*) MeOH in H_2_O (500 mL each) to give six fractions, SF-7394 (2)-1-6. Fraction SF-7394 (2)-4 (307.6 mg) was subjected to silica gel CC (3.0 × 25 cm) and eluted with CH_2_Cl_2_-MeOH (70:1–2:1) to yield subfractions (SF-7394 (2)-4-1-12). The subfraction SF-7394(2)-4-5 (34.9 mg) was isolated by a RP prep-HPLC eluting with a gradient of 20–50% CH_3_CN in H_2_O over 30 min to give a pseurotin A (**5**, 10.0 mg, *t*_R_ = 20.1 min). Subfraction SF7394(2)-4-8 (26.4 mg) was also purified by using a RP prep-HPLC (60–100% MeOH in H_2_O (0.1% HCOOH) over 50 min) to yield compound **2** (4.2 mg, *t*_R_ = 20.2 min). SF-7394 (2)-5 (786.7 mg) was first separated into five subfractions SF-7394 (2)-5-1~5 by using Sephadex LH-20 as the stationary phase and a 1/3 (*v*/*v*) mixture of MeOH in CHCl_3_ as the mobile phase on column (3.0 × 35 cm). The subfraction SF-7394 (2)-5-2 (20 mg) was further separated on a RP prep-HPLC (40–100% CH_3_CN in H_2_O (0.1% HCOOH) over 30 min) to provide two compounds, [D-Leu]-ternatin (**4**, 2.0 mg, *t*_R_ = 20.0 min) and (−)-ternatin (**3**, 10.1 mg, *t*_R_ = 21.0 min). Similarly, the subfraction SF-7394 (2)-5-3 (256 mg) was also purified by using a RP prep-HPLC (40–100% CH_3_CN in H_2_O (0.1% HCOOH) over 30 min) to yield compound **1** (4.6 mg, *t*_R_ = 33.8 min).

Acrepseudoterin (**1**): white amorphous solid; [α]^20^_D_ −15.6 (*c* 0.16, CH_3_CN); ECD (*c* 0.25 mM, CH_3_CN) *λ*_max_ (Δε) 233 (−10.3), 250 (+4.8); UV (MeOH) λ_max_ (log ε) 215 (3.86), 269 (3.02) nm; IR (ATR) ν_max_ 3280, 2923, 2866, 1646, 1590, 1458, 1379, 1049, 1016, 822 cm^−1^; ^1^H NMR and ^13^C NMR (400 and 100 MHz, pyridine-*d*_5_) see Table 1; HRESIMS *m*/*z* 309.2196 [M + Na]^+^ (calcd. for C_20_H_30_ONa, 309.2194).

Isocordycepoloside A (**2**): colorless gum; [α]^20^_D_ +18.6 (*c* 0.42, CH_3_OH); IR (ATR) ν_max_ 3372, 2952, 2871, 1707, 1670, 1446, 1380, 1284, 1145, 1056, 1023, 944, 920, 878, 832 cm^−1^; ^1^H NMR and ^13^C NMR (400 and 100 MHz, pyridine-*d*_5_) see Table 2; HRESIMS *m*/*z* 421.2213 [M + Na]^+^ (calcd. for C_21_H_34_O_7_Na, 421.2202).

### 3.4. Determination of the Glucose Configuration

Compound **2** (1.0 mg) was dissolved in of 2 M HCl (200 μL) and heated at 90 °C for 2 h in a screw-capped vial. After hydrolysis, the reaction mixture was neutralized with 2 M NH_4_OH (220 μL) and dried in vacuo.

Each monosaccharide or acid hydrolysate of **1** (1.0 mg) and D/L-cysteine methyl ester (1.0 mg) were dissolved in pyridine (200 μL) and heated at 60 °C for 1 h in a screw-capped vial. Then, phenyl isothiocyanate (5 μL) was added to the mixture and heated further for 1 h. The reaction mixture (5 μL) was further diluted 5 times before HPLC analysis and detected at 250 nm with a photodiode array detector. Analytical HPLC was carried out with the isocratic elution of 25% CH_3_CN in 50 mM H_3_PO_4_ for 40 min, and the subsequent washing of the column was performed with 90% CH_3_CN at a flow rate of 0.8 mL/min on a Phenomenex Gemini NX-C_18_ (4.6 × 250 mm; 5 μm). The retention times (*t*_R_) of the derivative prepared from standard D-glucose and L-cysteine methyl ester, and the derivative prepared from standard L-glucose and L-cysteine methyl ester, were 38.0 and 36.9 min, respectively. The retention time of the derivative prepared from the acid hydrolysate of **1** and L-cysteine coincided with that obtained for the derivative of D-glucose.

### 3.5. Computational Methods

The conformer distribution was conducted using an MMFF force field with Spartan ′14 software (Wave-function, Inc., Irvine, CA, USA). The geometry optimization for selected conformers was performed at the DFT (B3LYP functional/6-31+G(d,p) basis set) level, and ECD calculations were performed at the TDDFT (CAM-B3LYP/SVP basis set) level with a CPCM solvent model in CH_3_CN using Gaussian 09 software (Gaussian, Inc., Wallingford, CT, USA). The calculated ECD curves were simulated by SpecDis 1.64 software (University of Wuerzburg, Wuerzburg, Germany) with a half bandwidth of 0.3 eV. The ECD curves of conformers were weighted using the Boltzmann distribution after UV correction.

### 3.6. PTP1B Inhibitory Activity Assay

PTP1B (human, recombinant) was purchased from ATGen Co., Ltd. (Seongnam-si, Korea). The enzyme activity was measured in a reaction mixture containing 1 mM *p*-NPP in 50 mM Bis-Tris (pH 6.0), 2 mM EDTA, and 5 mM dithiothreitol (DTT). After incubation at 37.5 °C for 30 min, the reaction was terminated by the addition of 10 N NaOH. The amount of *p*-nitrophenol induced was determined by measuring the increase in absorbance at 405 nm. The non-enzymatic hydrolysis of 1 mM *p*-NPP was corrected by measuring the increase in absorbance at 405 nm obtained in the absence of the PTP1B enzyme. The kinetic analysis of PTP1B inhibition was conducted in the presence or absence of compound **1** with different concentrations of *p*-NPP as a PTP1B substrate. The Michaelis–Menten constant (*K*_m_) and maximum velocity (*V*_max_) of PTP1B were determined by the Lineweaver–Burk plot using a Graph Pad Prism^®^ 4 software (Graph Pad Software Inc., San Diego, CA, USA).

## 4. Conclusions

In this study, two new terpenoid-type fungal metabolites named acrepseudoterin (**1**) and isocordycepoloside A (**2**) were isolated from extracts of the Antarctic lichen-derived fungal strain *Acremonium* sp. SF-7394 along with three previously described fungal metabolites. The structure elucidation of these fungal metabolites was carried out mainly by analysis of their NMR and MS data, and the absolute configurations of **1** and **2** were assigned by electronic circular dichroism calculations and a chemical method. In the biological evaluation of these metabolites, acrepseudoterin (**1**) and [D-Leu]-ternatin (**4**) were identified as mild inhibitors of protein tyrosine phosphatase 1B (PTP1B), which is considered as the drug target for the treatment of type 2 diabetes mellitus and obesity. Taken together, this study supports the hypothesis that the fungal metabolites from extreme environments including the Antarctic area could serve as valuable resources for biologically active compounds with novel structures [20].

## Figures and Tables

**Table 1:** NMR spectroscopic data (400 MHz, pyridine-*d*_5_) for Compound **1**.

Position	δ_C_, Type	δ_H_ (*J* in Hz)	HMBC	NOESY
1	32.9, CH	3.35, m		4
2	34.7, CH_2_	1.85, dt (2.2, 13.2) 1.46, m	1, 3, 4, 12	
3	29.1, CH	1.68, m		
4	45.6, CH	2.10, m	3, 6, 13, 18	1, 7
5	28.7, CH_2_	α 2.15, m β 1.25, m		18, 19
6	33.0, CH_2_	2.04, m 1.41, m		
7	34.6, CH	2.98, m	6, 8, 9, 13, 19	4
8	141.2, C			
9	126.2, CH	7.25, d (7.8)	7, 11, 13	
10	126.9, CH	7.79, d (7.8)	8, 12, 20	
11	138.1, C			
12	139.4, C			
13	136.4, C			
14	46.4, CH_2_	1.57, td (3.4, 8.0) 1.33, m		
15	26.1, CH	1.72 m		
16	21.2, CH_3_	1.00, d (6.4)	14, 17	
17	24.8, CH_3_	0.85, d (6.4)	14, 15, 16	
18	21.5, CH_3_	1.03, d (6.4)	2, 3, 4	5α
19	24.4, CH_3_	1.31, d (6.6)	6, 8	5α
20	62.1, CH_2_	5.18, dd (3.2, 12.9) 5.03, dd (3.2, 12.9)		
20-OH		6.66, br t		

**Table 2:** NMR spectroscopic data (400 MHz, pyridine-*d*_5_) for compound **2**.

Position	δ_C_, Type	δ_H_ (*J* in Hz)	HMBC	NOESY
1	52.3, CH	1.65, m	2, 5, 6, 10	6, 13, 14
2	21.0, CH_2_	2.01, m 1.76, m	1, 4, 5	
3	29.4, CH_2_	1.86, m 1.00, m		
4	34.8, CH	1.97, m	1, 3, 5, 10, 14	
5	43.5, C	-	-	
6	77.6, CH	3.51, d (5.6)	1, 5, 7, 8, 10, 12	1, 12β, 14
7	121.5, CH	5.71, brd (5.6)	5, 6, 9, 15	
8	141.2, C			
9	28.6, CH_2_	1.77, m 1.95, m	5, 7, 8	
10	23.3, CH_2_	1.26, m 2.93, ddd (6.8, 12.8, 18.5)	4, 5, 6, 8, 9	
11	76.5, C			
12	75.7, CH_2_	α 4.57, d (12.4) β 3.15, d (12.0)	1, 6, 11	13 13, 6
13	20.1, CH_3_	1.31, s	1, 11, 12	
14	18.6, CH_3_	0.76, d (6.8)	3, 4, 5	1, 6
15	23.4, CH_3_	1.47, s	7, 8, 9	
1′	95.4, CH	5.57, d (3.6)	11, 3′, 5′	
2′	74.1, CH	4.09, dd (3.6, 9.6)	3′	
3′	75.6, CH	4.58, t (9.6)	2′, 4′	
4′	72.1, CH	4.22, t (9.6)	5′, 6′	
5′	74.5, CH	4.69, m		
6′	62.9, CH_2_	4.40, dd (12.0, 2.0) 4.31, dd (11.6, 4.8)	4′	

## Data Availability

The data presented in this study are available in this article.

## Supplementary Materials

The following are available online. Figure S1: ^1^H-NMR spectrum of **1** in pyridine-*d*_5_. Figure S2: ^13^C-NMR spectrum of **1** in pyridine-*d*_5_. Figure S3: HMQC spectrum of **1** in pyridine-*d*_5_. Figure S4: COSY spectrum of **1** in pyridine-*d*_5_. Figure S5: HMBC spectrum of **1** in pyridine-*d*_5_. Figure S6: NOESY spectrum of **1** in pyridine-*d*_5_. Figure S7: Zoomed-in NOESY spectrum of **1**. Figure S8: Zoomed-in NOESY spectrum of **1** with increased intensity. Figure S9: HRESIMS of **1**. Figure S10: ^1^H-NMR spectrum of **2** in pyridine*-d*_5_. Figure S11: ^13^C-NMR spectrum of **2** in pyridine*-d*_5_. Figure S12: HMQC spectrum of **2** in pyridine*-d*_5_. Figure S13: COSY spectrum of **2** in pyridine*-d*_5_. Figure S14: HMBC spectrum of **2** in pyridine*-d*_5_. Figure S15: NOESY spectrum of **2** in pyridine*-d*_5_. Figure S16: Zoomed-in NOESY spectrum of **2**. Figure S17: HRESIMS of **2**. Figure S18: ^1^H-NMR spectrum of **3** in benzene-*d*_6_. Figure S19: ^13^C-NMR spectrum of **3** in benzene-*d*_6_. Figure S20: HRESIMS of **3**. Figure S21: ^1^H-NMR spectrum of **4** in benzene-*d*_6_. Figure S22: HRESIMS of **4**. Figure S23: ^1^H-NMR spectrum of **5** in DMSO-*d*_6_. Figure S24: ^13^C-NMR spectrum of **5** in DMSO-*d*_6_. Figure S25: HRESIMS of **5**. Figure S26: HPLC traces of the thiocarbamoyl-thiazolidine derivatives L and D-glucose, and the hydrolizate of **2**.

## Sample Availability

Samples of the compounds **1**–**5** are available from the authors.
