# Supplementary material for: PTP1B Inhibitory Secondary Metabolites from an Antarctic Fungal Strain Acremonium sp. SF-7394
Source: Molecules. 2021 Sep 10;26(18):5505. doi: 10.3390/molecules26185505 (PMC8468024; doi:10.3390/molecules26185505)
Supplement: Supplementary file 1 [file molecules-26-05505-s001.zip › molecules-1340986-supplementary.pdf]

Supplementary Materials

PTP1B Inhibitory Secondary Metabolites from an Antarctic Fungal Strain *Acremonium* sp. SF-7394

Hye Jin Kim [1,†], Xiao-Jun Li [1,†], Dong-Cheol Kim [1], Tai Kyoung Kim [2], Jae Hak Sohn [3], Haeun Kwon [4], Dongho Lee [4], Youn-Chul Kim [1], Joung Han Yim [2,*] and Hyuncheol Oh [1,*]

1 Institute of Pharmaceutical Research and Development, College of Pharmacy, Wonkwang University, Iksan 54538, Korea; mn1003@naver.com (H.J.K.); lixiaojun2017@yahoo.com (X.-J.L.); kimman07@hanmail.net (D.-C.K.); yckim@wku.ac.kr (Y.-C.K.)

2 Division of Polar Life Sciences, Korea Polar Research Institute, Incheon 21990, Korea; tkkim@kopri.re.kr

3 College of Medical and Life Sciences, Silla University, Busan 46958, Korea; jhsohn@silla.ac.kr

4 Department of Plant Biotechnology, College of Life Sciences and Biotechnology, Korea University, Seoul 02841, Korea; haeun9906@daum.net (H.K.); dongholee@korea.ac.kr (D.L.)

* Correspondence: jhyim@kopri.re.kr (J.H.Y.); hoh@wku.ac.kr (H.O.); Tel.: +82-63-850-6815 (H.O.)

† These authors contributed equally to this work.

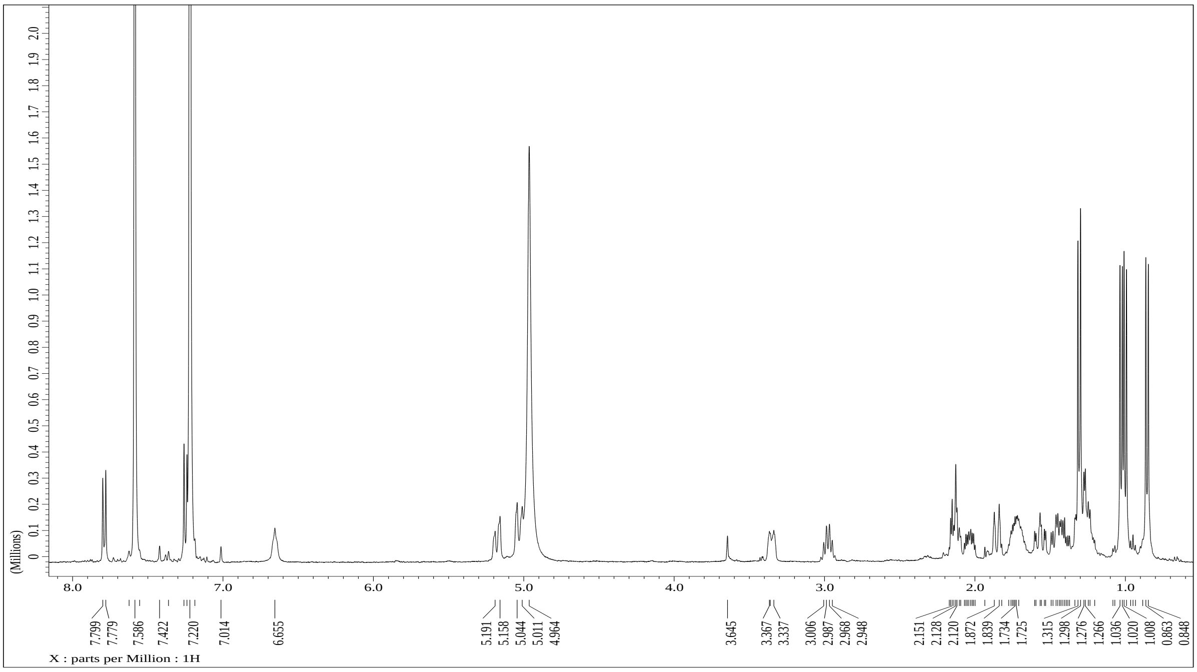

Figure S1. ^{1}H-NMR spectrum of **1** in pyridine-*d*$_5$

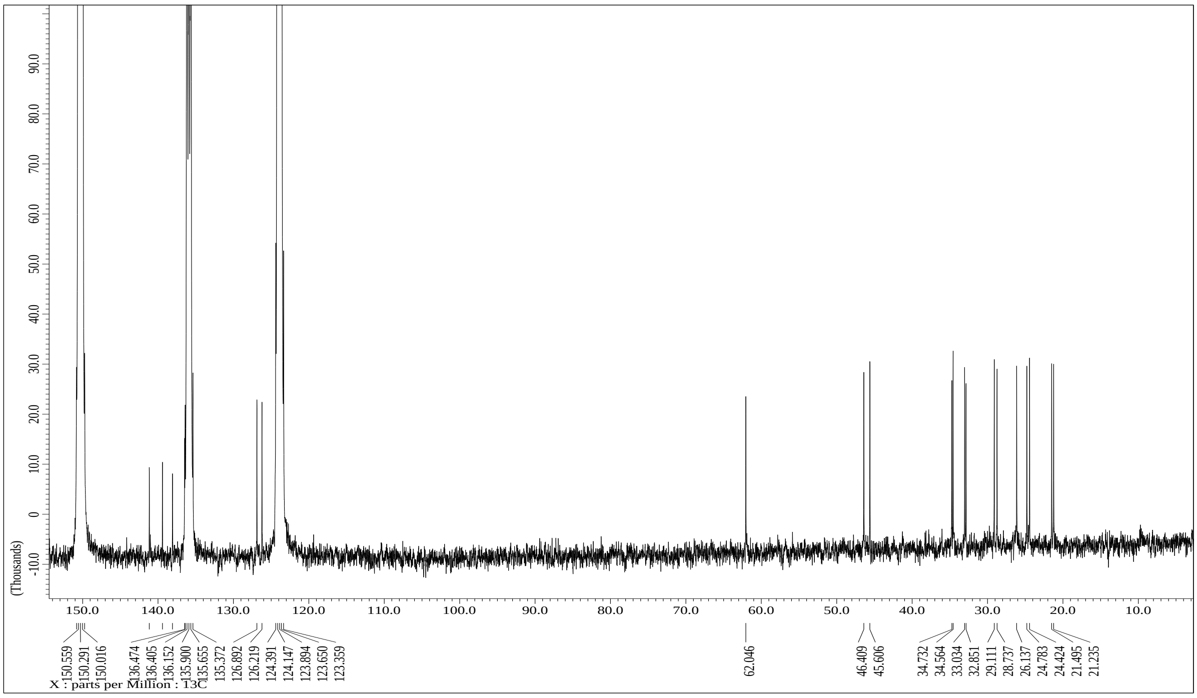

Figure S2. ^{13}C-NMR spectrum of **1** in pyridine-*d*$_5$

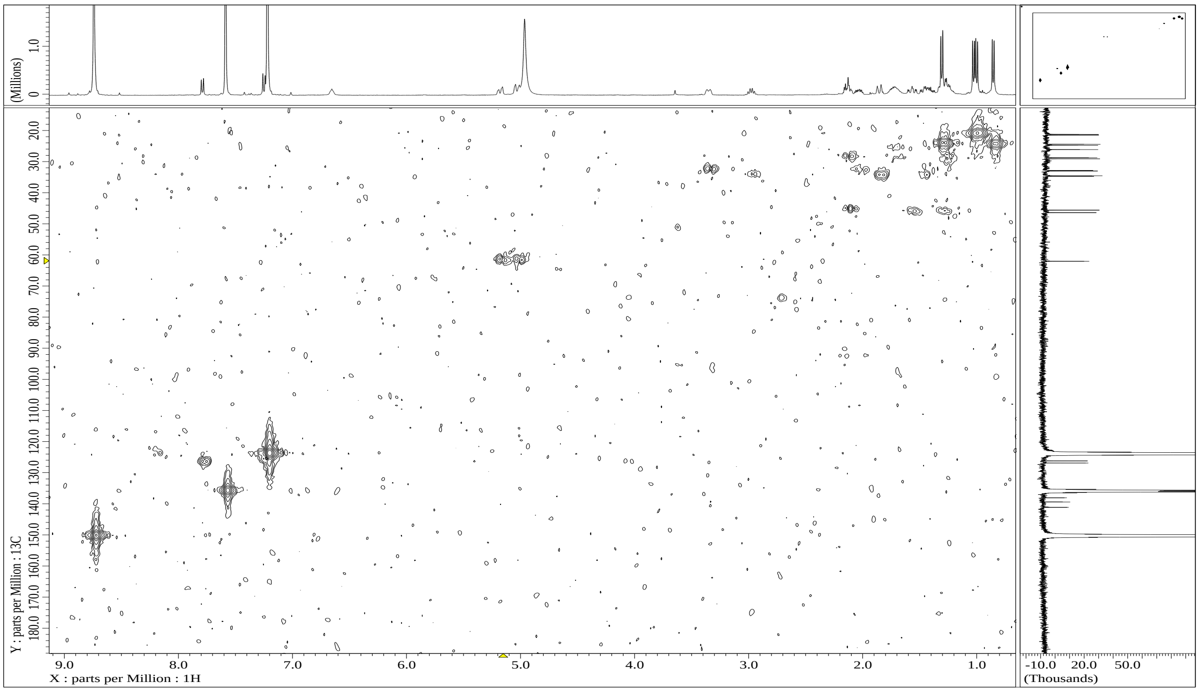

Figure S3. HMQC spectrum of **1** in pyridine-d_5

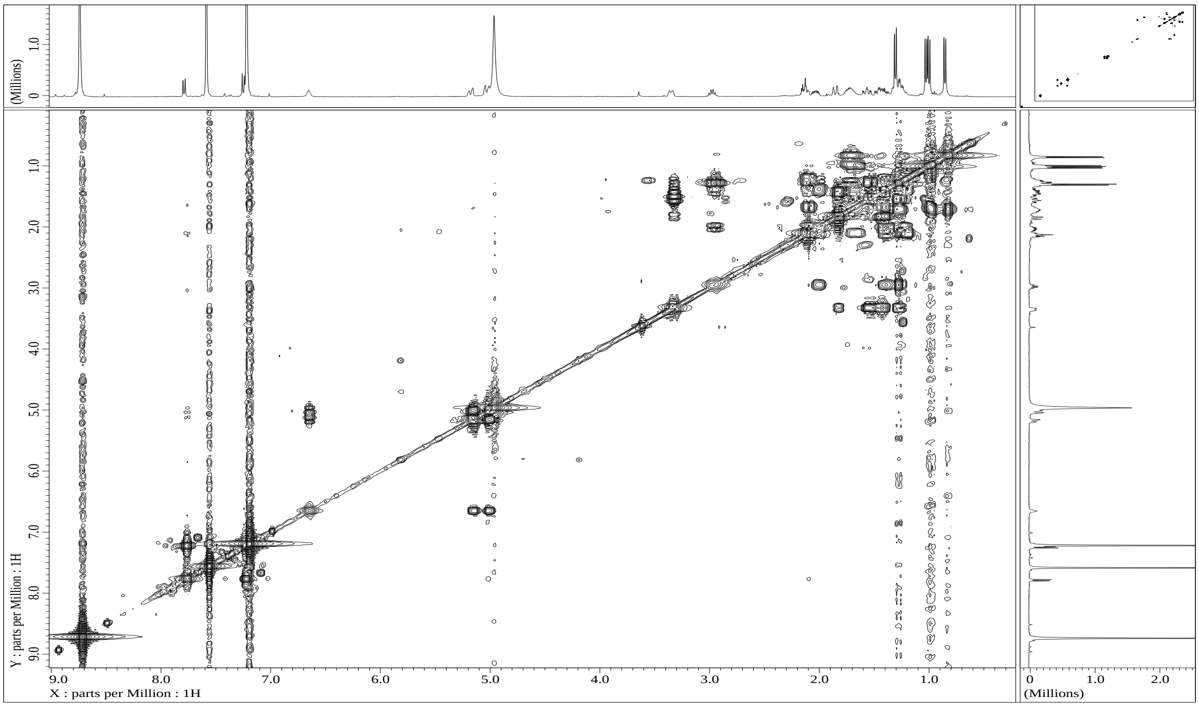

Figure S4. COSY spectrum of **1** in pyridine-d_5

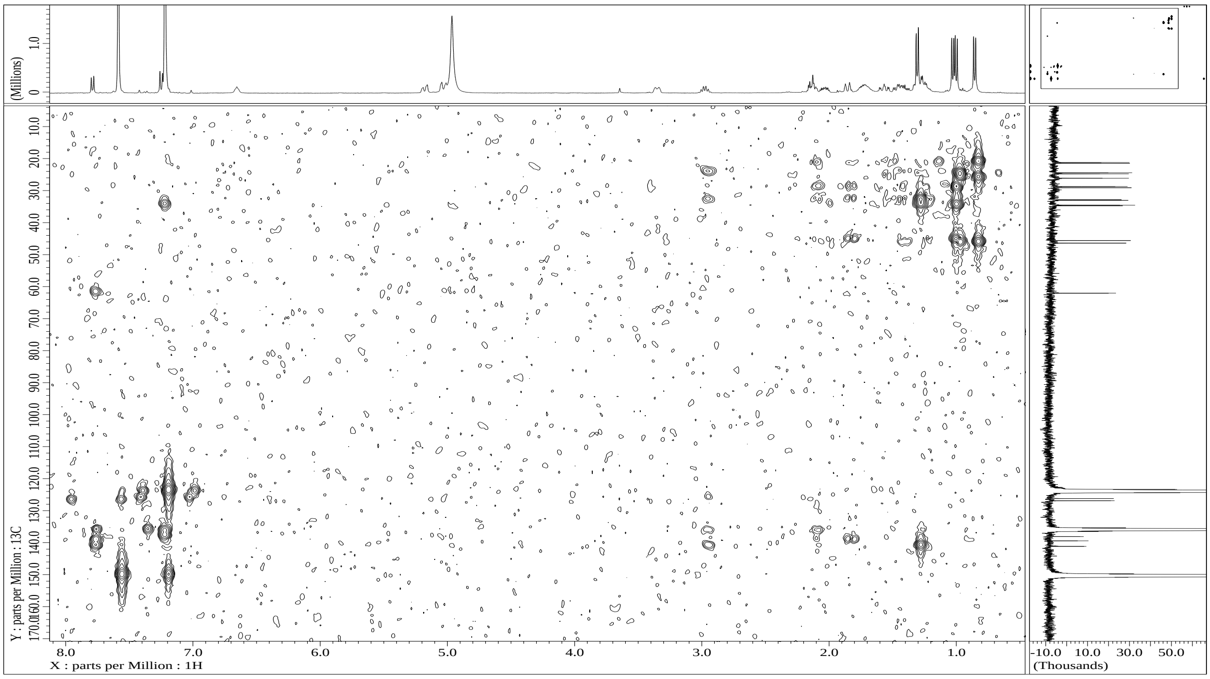

Figure S5. HMBC spectrum of **1** in pyridine-d_5

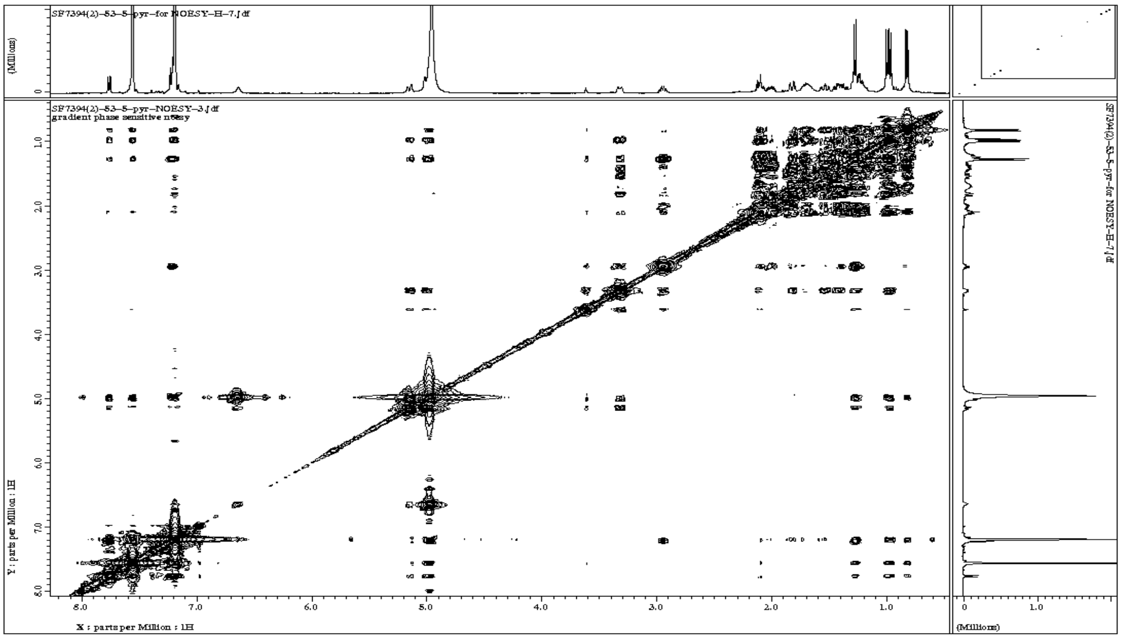

Figure S6. NOESY spectrum of **1** in pyridine-d_5

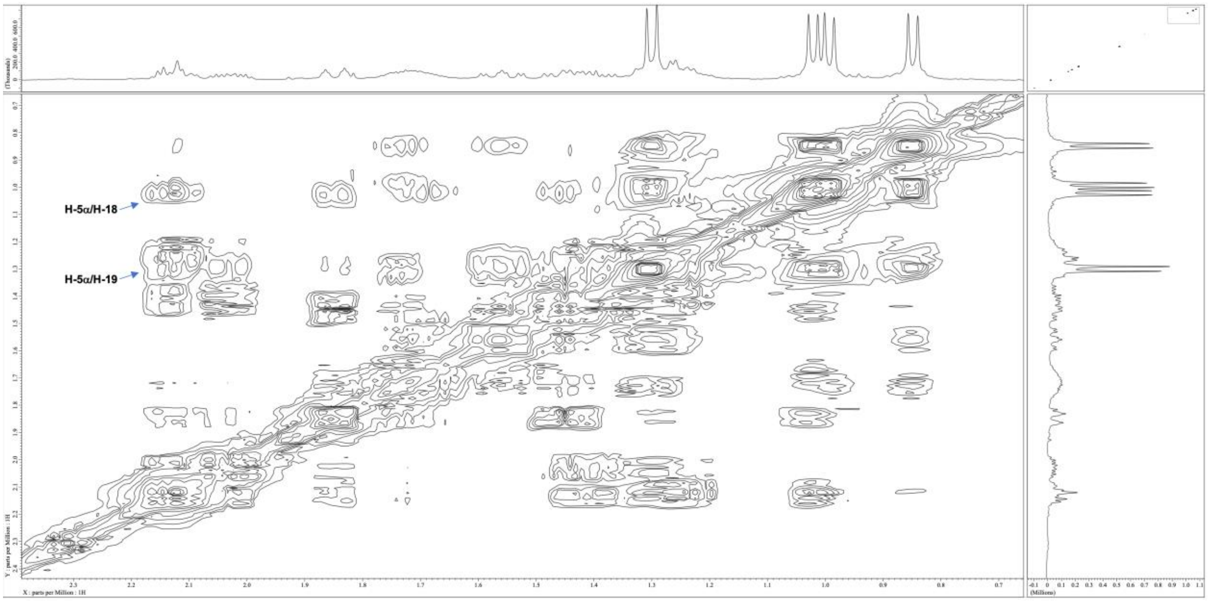

Figure S7. Zoomed-in NOESY spectrum of **1**

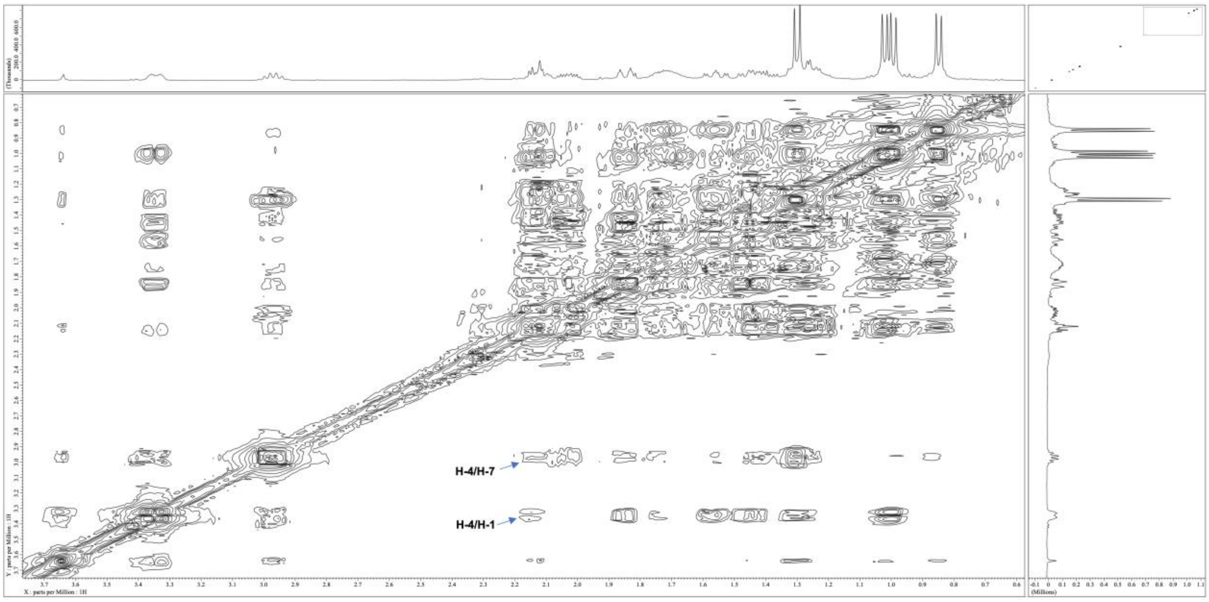

Figure S8. Zoomed-in NOESY spectrum of **1** with increased intensity

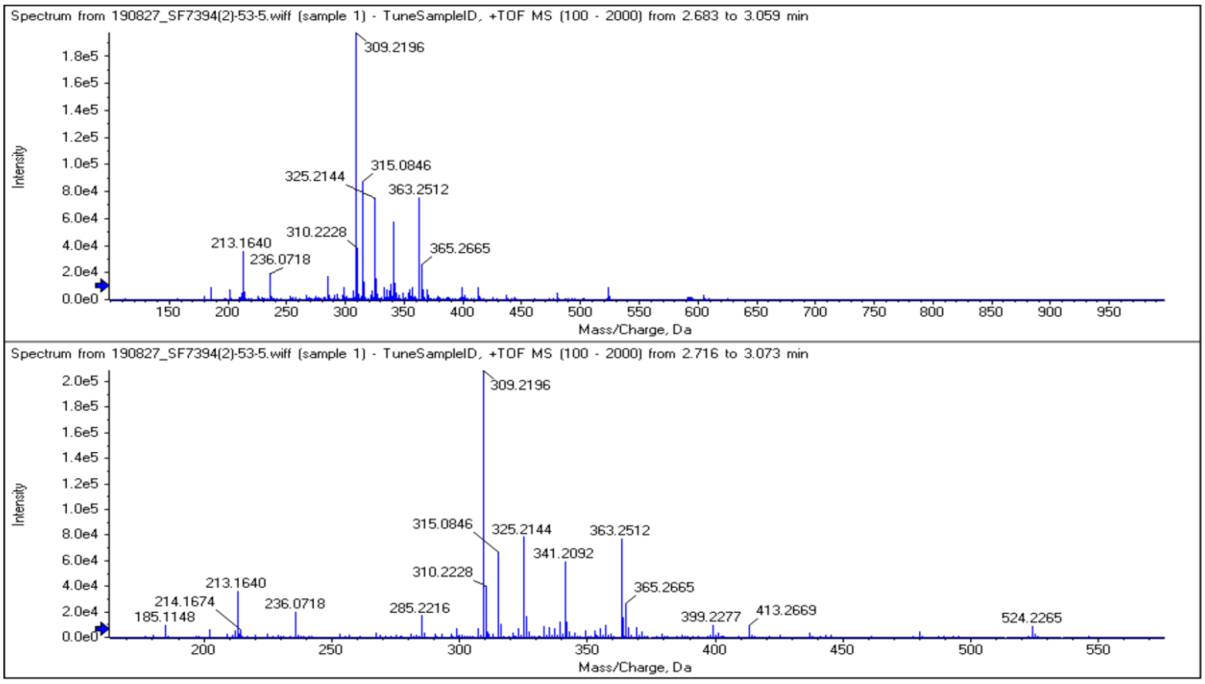

Figure S9. HRESIMS of **1**

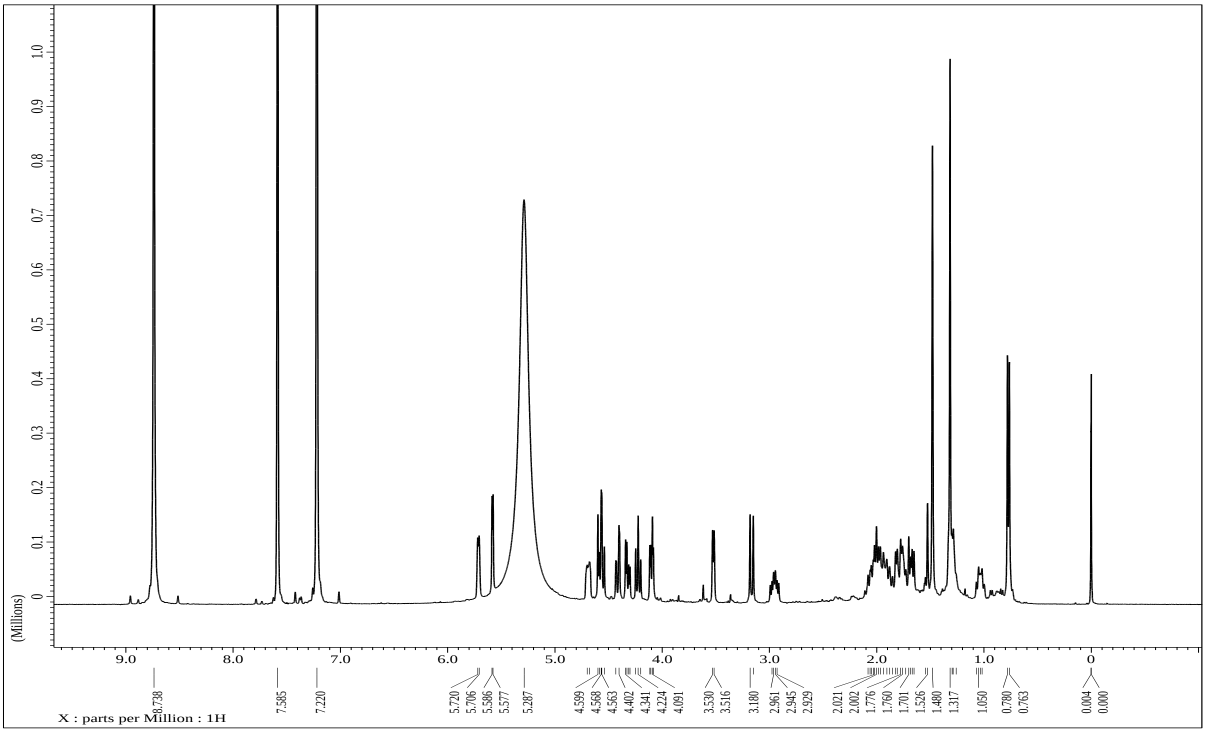

Figure S10. ^{1}H-NMR spectrum of **2** in pyridine-d_5

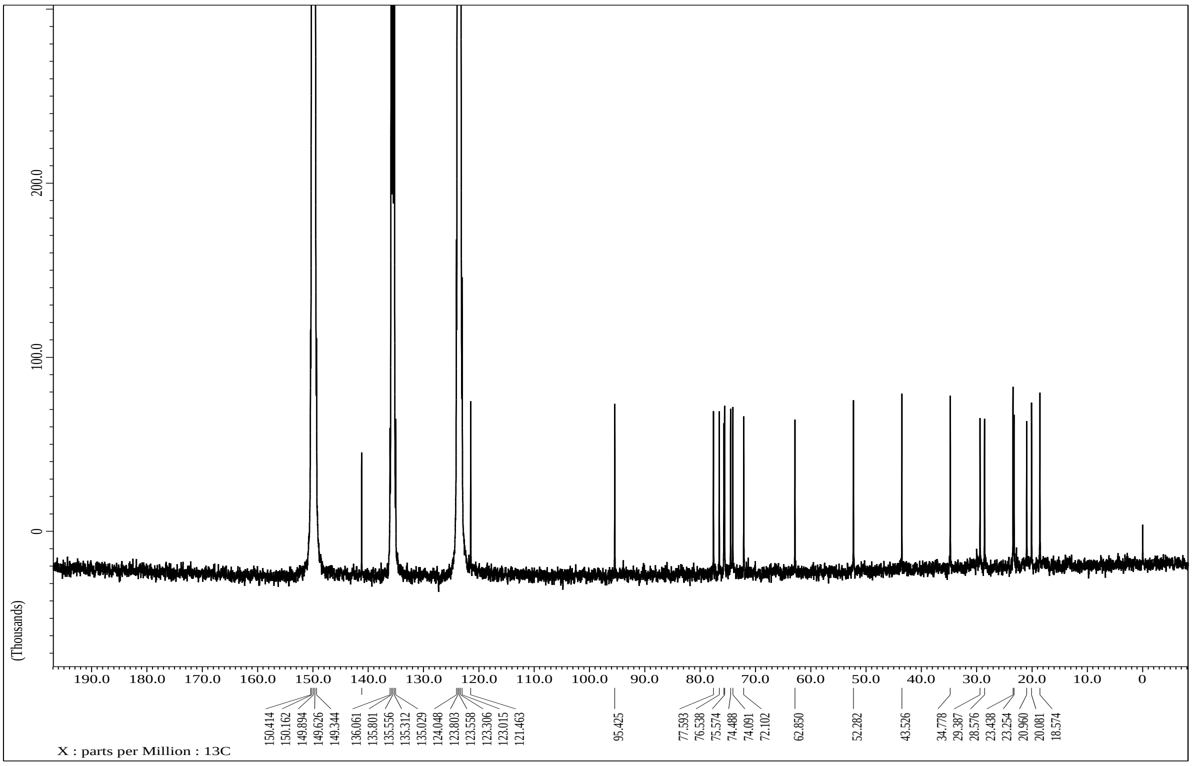

Figure S11. ^{13}C-NMR spectrum of **2** in pyridine-d_5

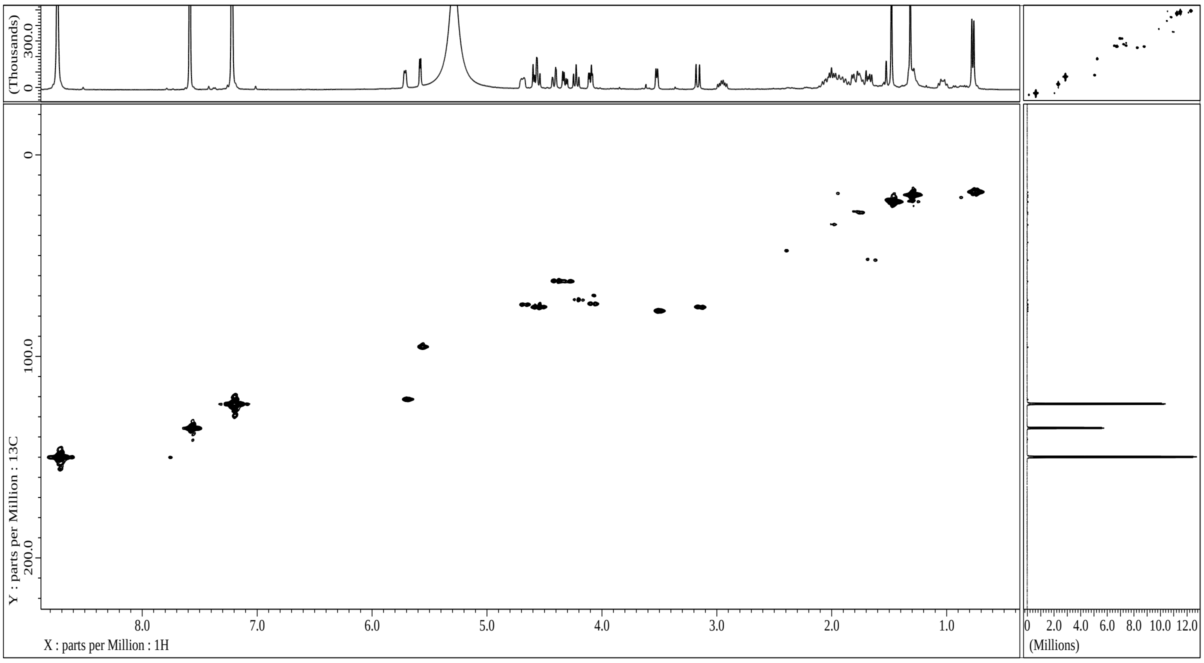

Figure S12. HMQC spectrum of **2** in pyridine-d_5

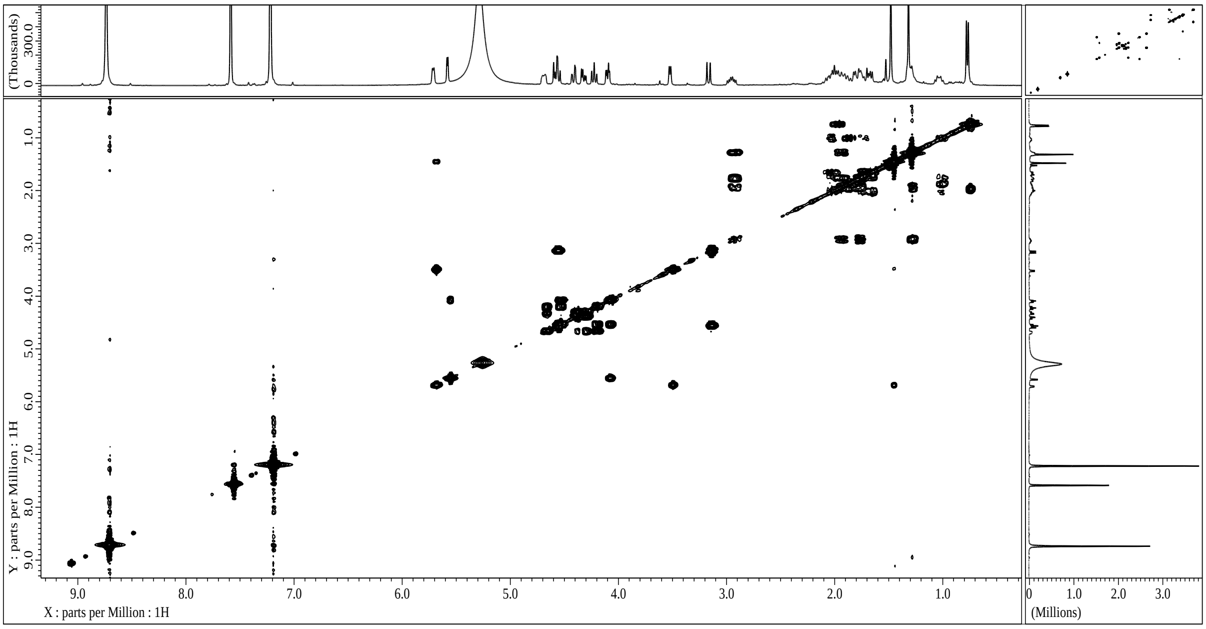

Figure S13. COSY spectrum of **2** in pyridine-*d*5

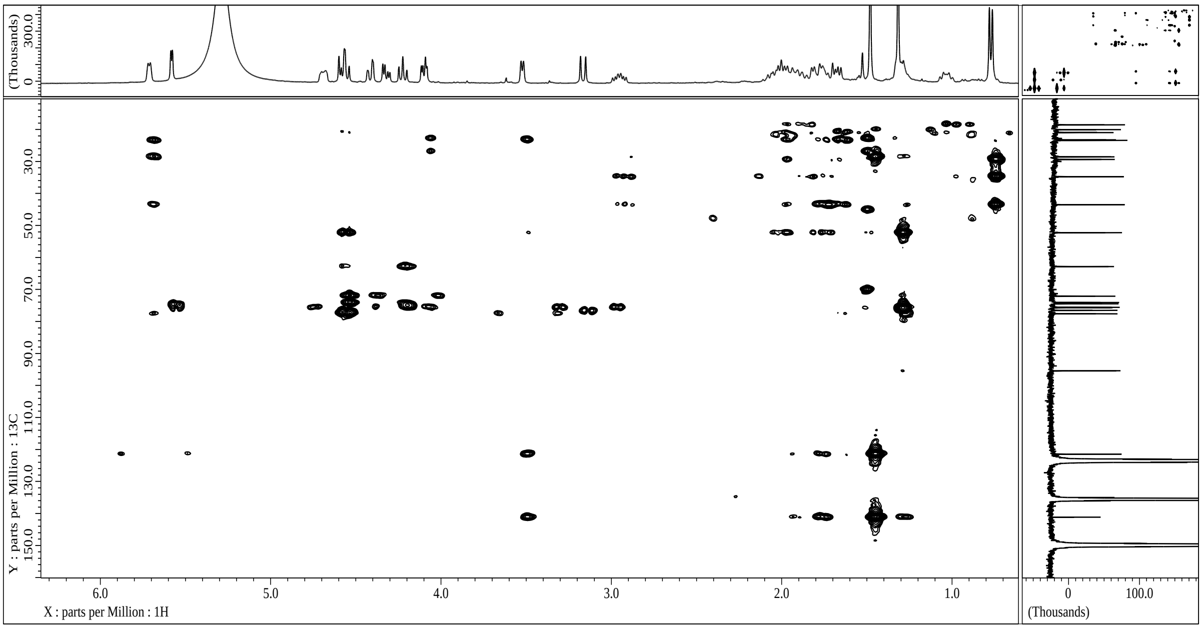

Figure S14. HMBC spectrum of **2** in pyridine-*d*5

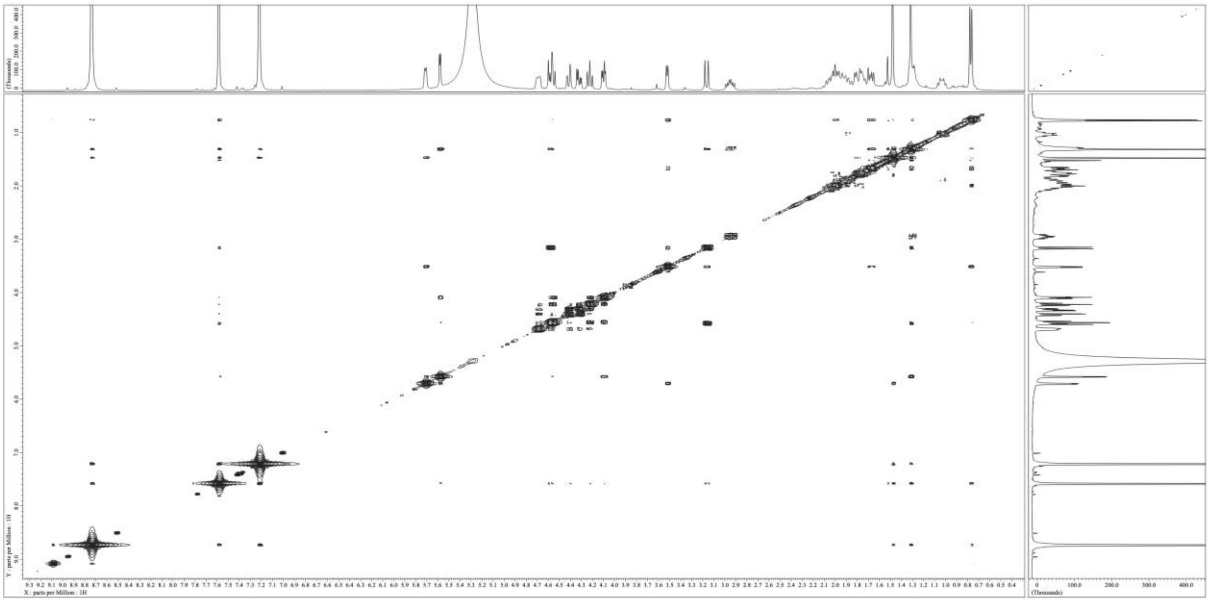

Figure S15. NOESY spectrum of **2** in pyridine-d_5

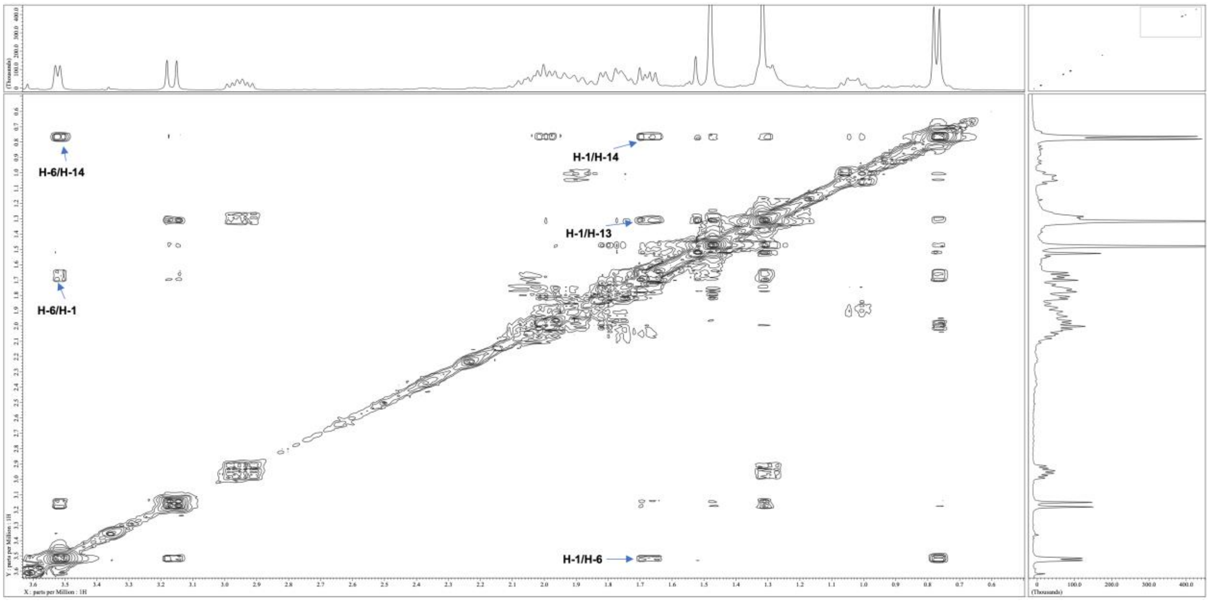

Figure S16. Zoomed-in NOESY spectrum of **2**

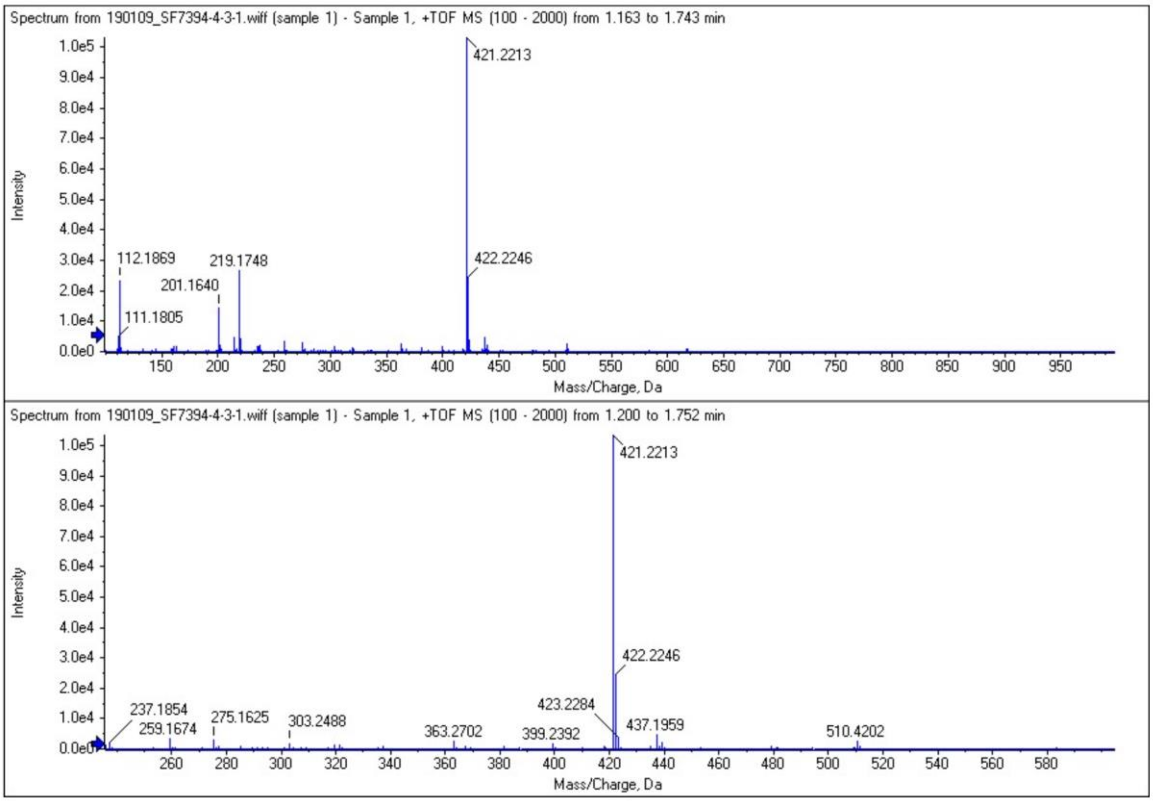

Figure S17. HRESIMS of **2**

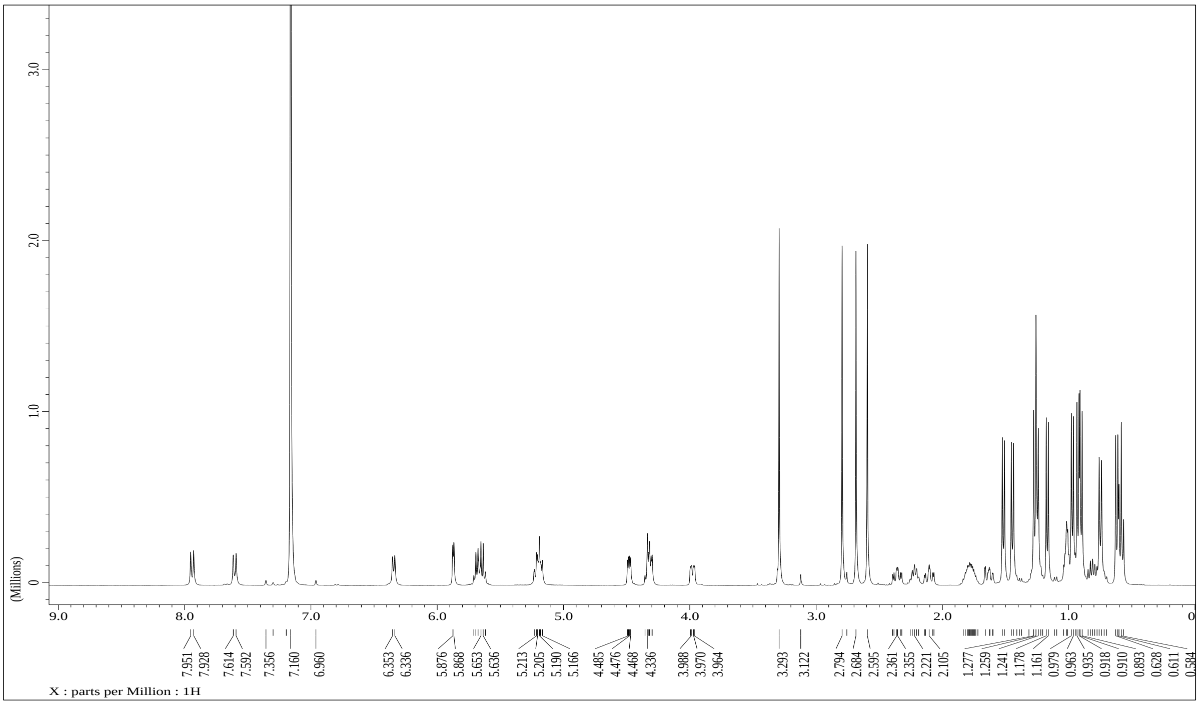

Figure S18. ^{1}H-NMR spectrum of **3** in benzene-d_6

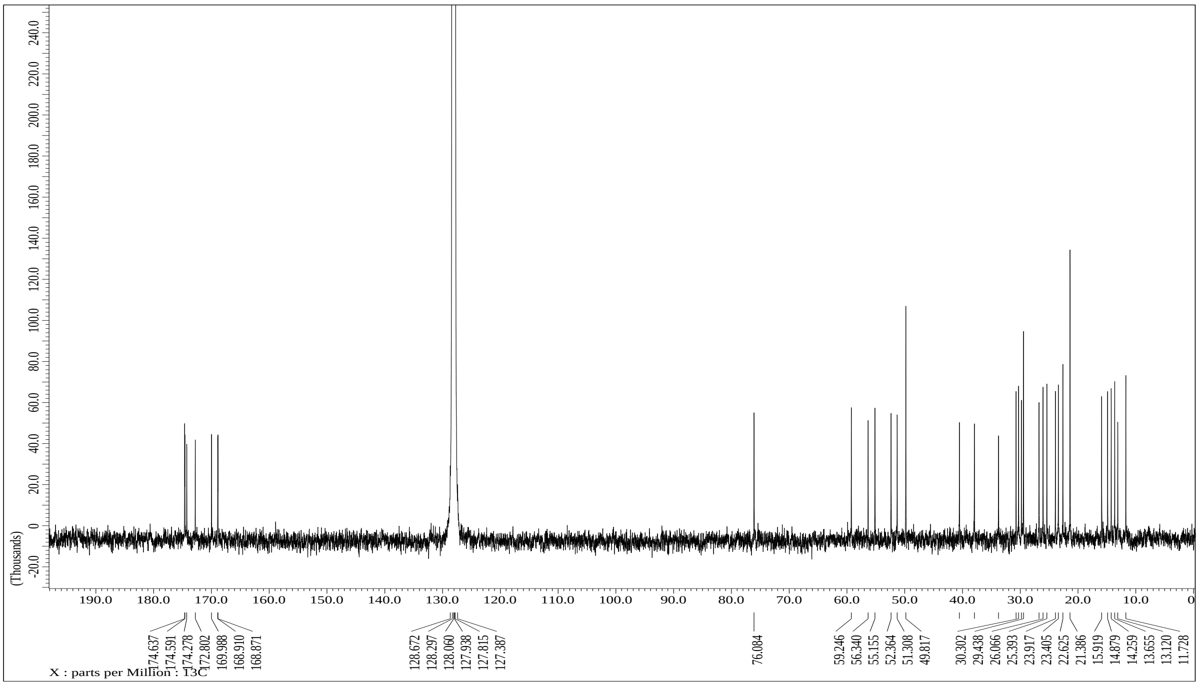

Figure S19. ^{13}C-NMR spectrum of **3** in benzene-d_6

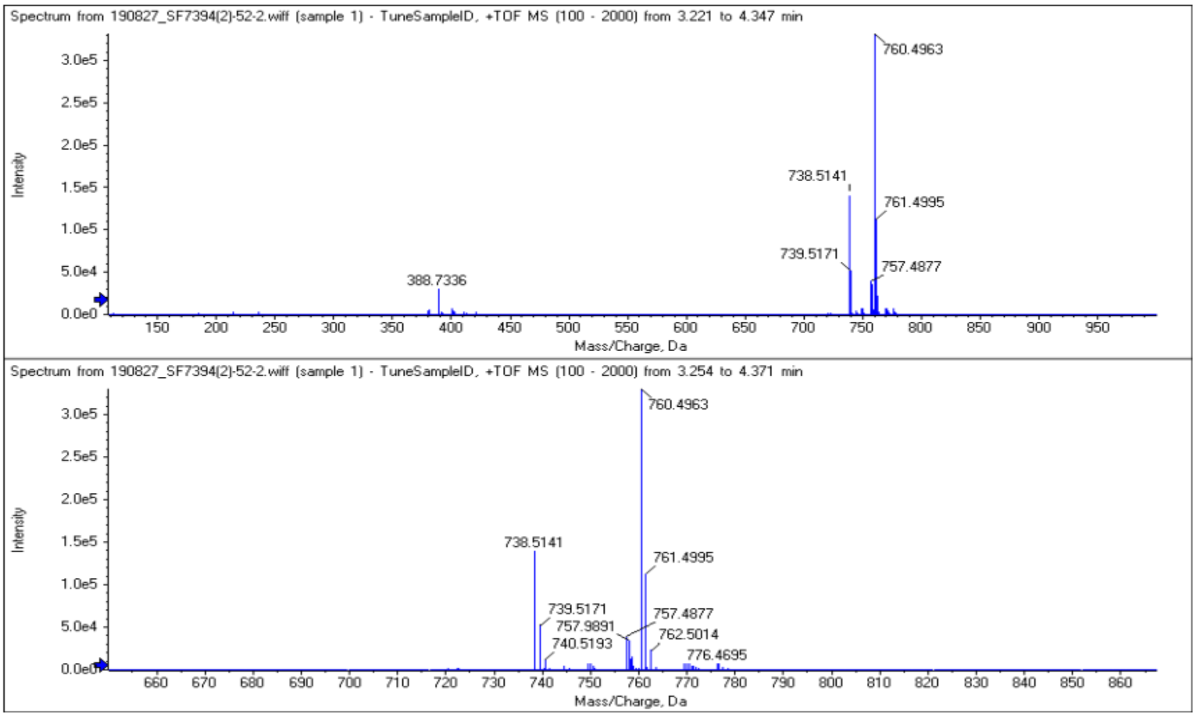

Figure S20. HRESIMS of **3**

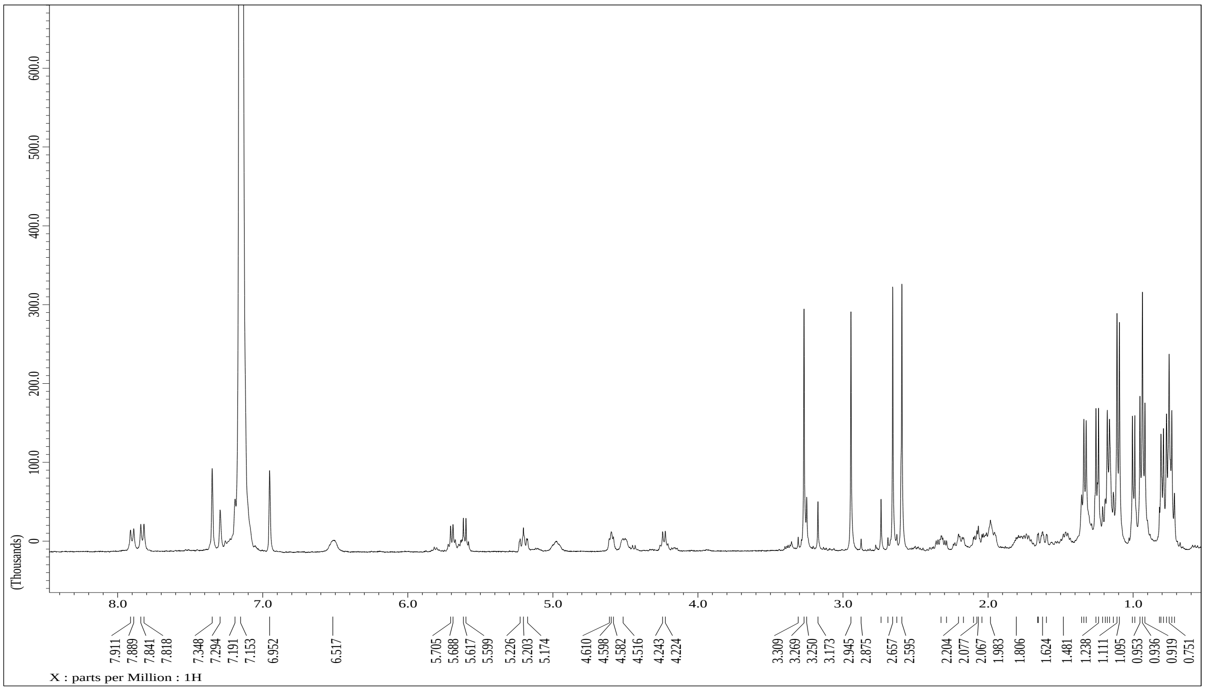

Figure S21. ^{1}H-NMR spectrum of **4** in benzene-d_6

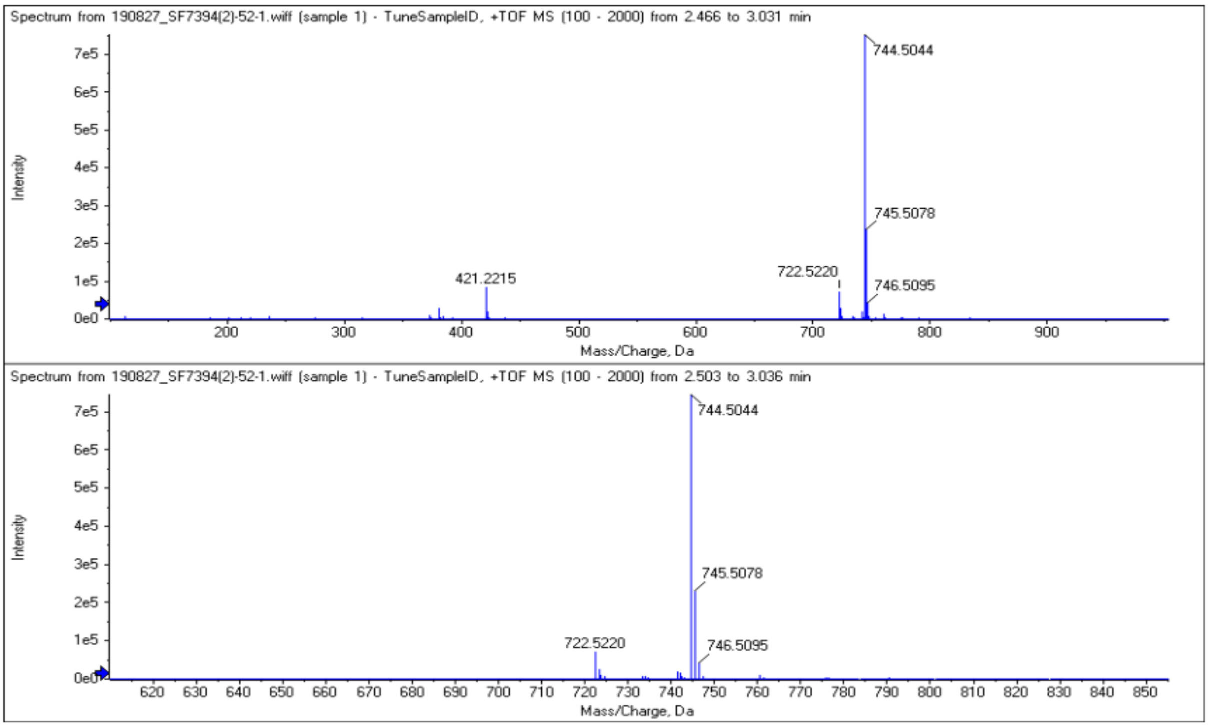

Figure S22. HRESIMS of **4**

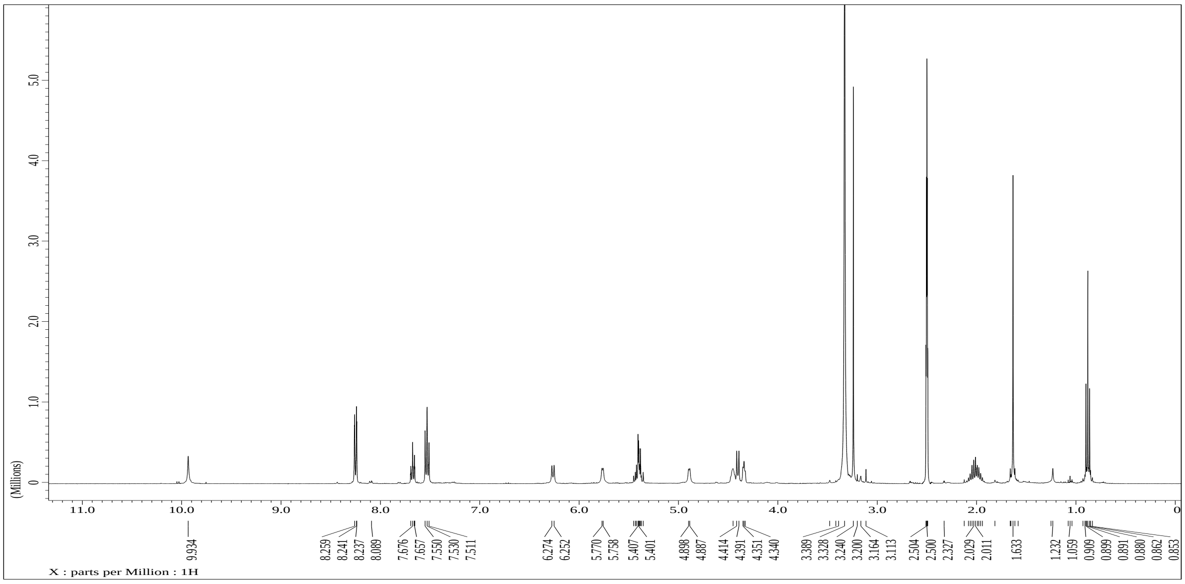

Figure S23. ^{1}H-NMR spectrum of **5** in DMSO-d_6

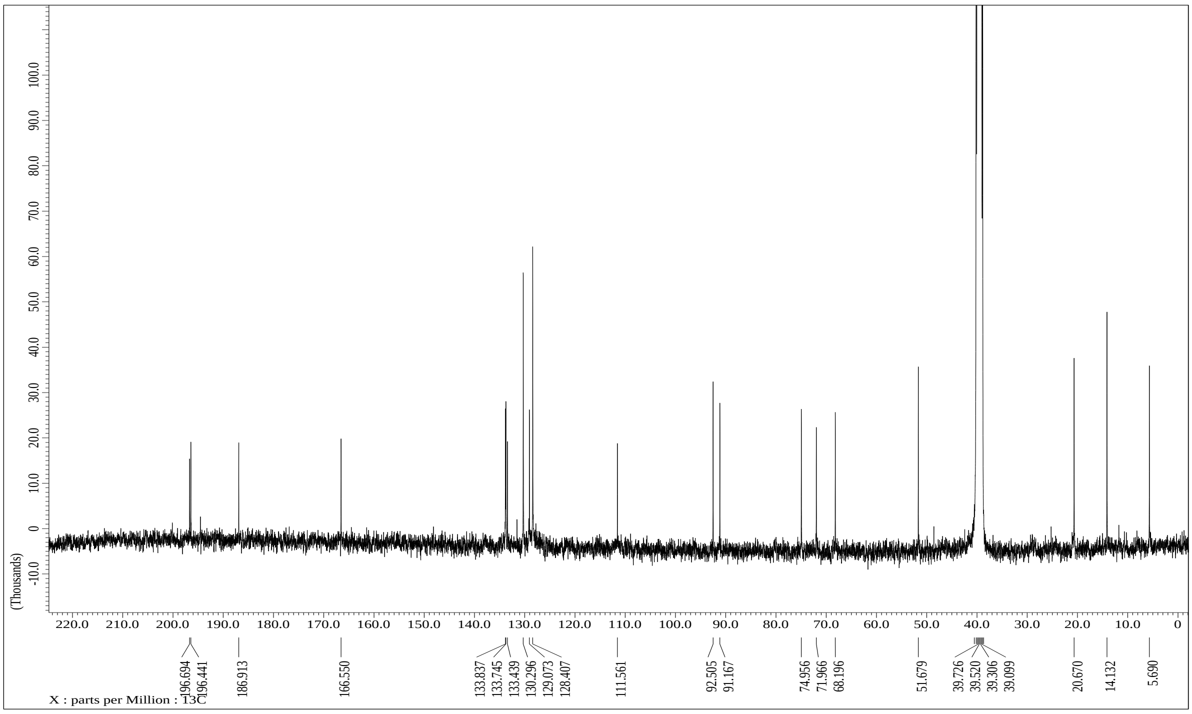

Figure S24. ^{13}C-NMR spectrum of **5** in DMSO-d_6

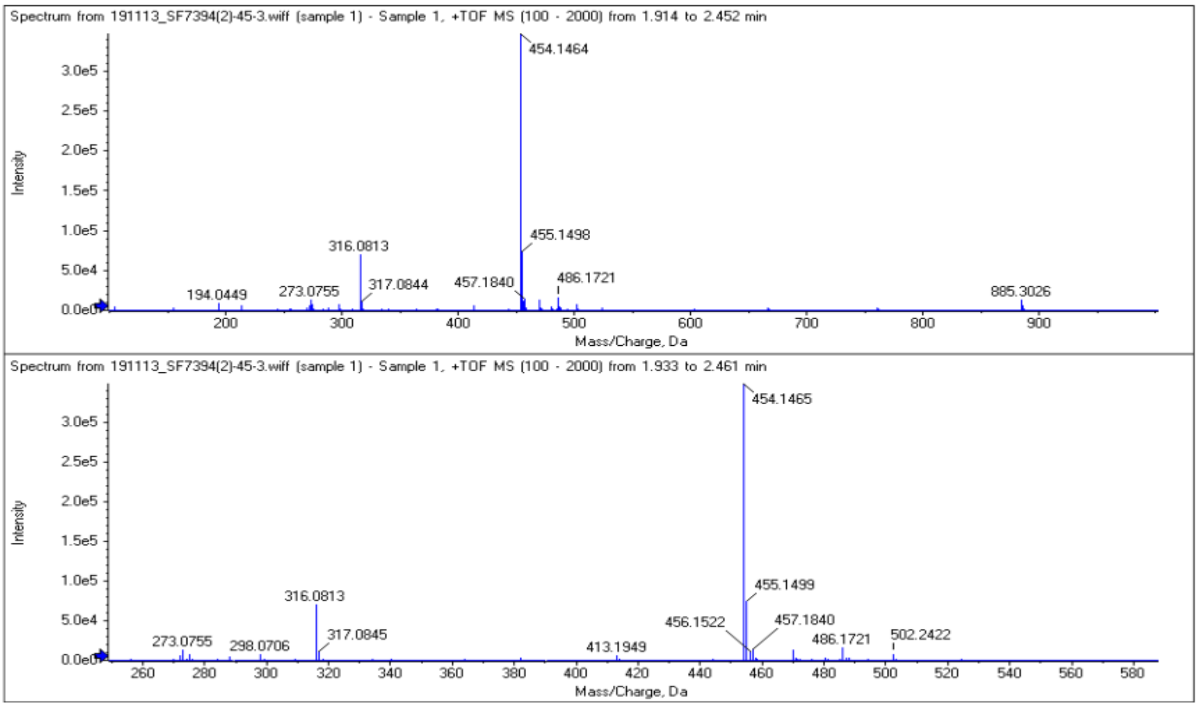

Figure S25. HRESIMS of **5**

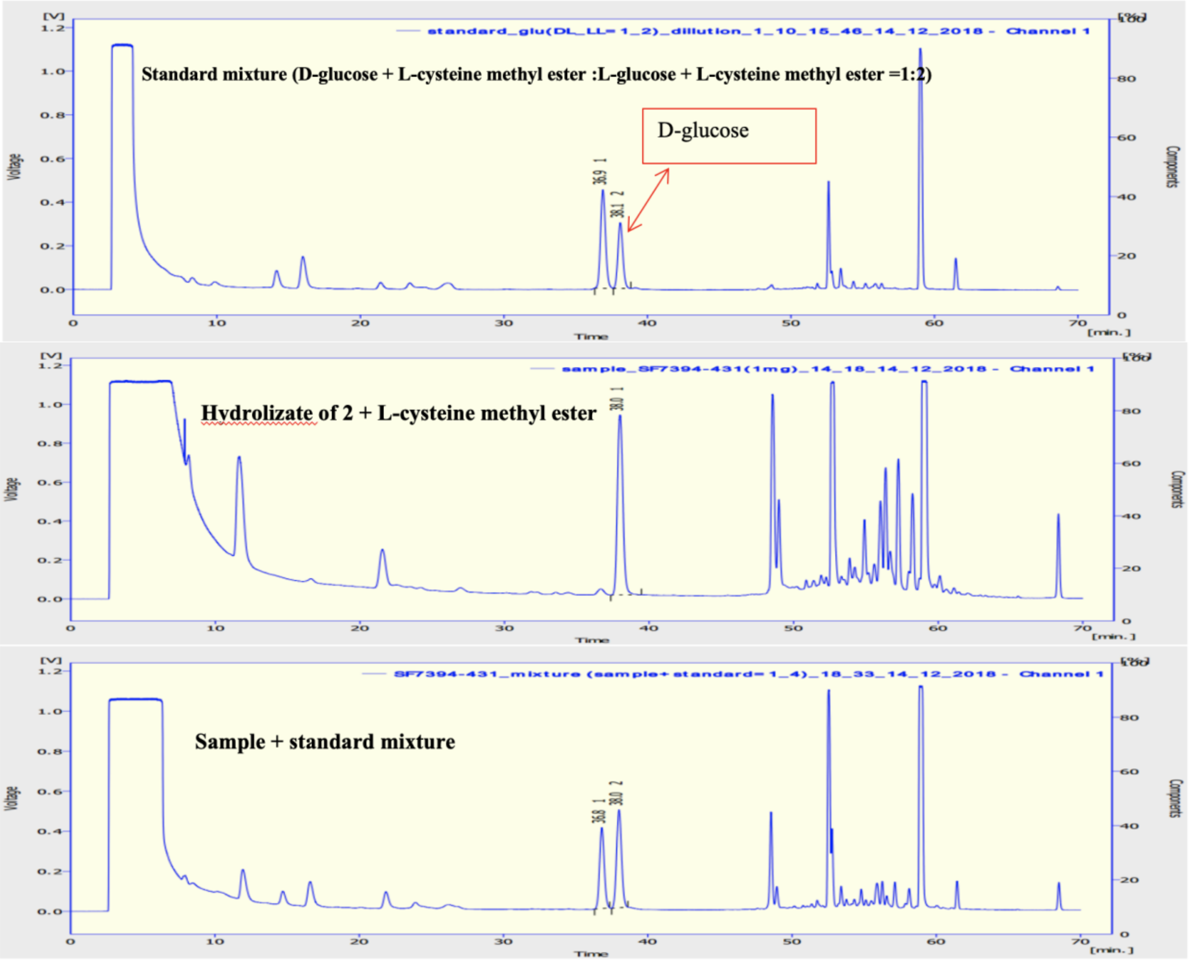

Figure S26. HPLC traces of the thiocarbamoyl-thiazolidine derivatives L and D- glucose, and the hydrolizate of **2**
